# Fruit metabolite networks in engineered and non-engineered tomato genotypes reveal fluidity in a hormone and agroecosystem specific manner

**DOI:** 10.1007/s11306-016-1037-2

**Published:** 2016-05-11

**Authors:** Tahira Fatima, Anatoly P. Sobolev, John R. Teasdale, Matthew Kramer, Jim Bunce, Avtar K. Handa, Autar K. Mattoo

**Affiliations:** Sustainable Agricultural Systems Laboratory, United States Department of Agriculture, Agricultural Research Service, The Henry A. Wallace Beltsville Agricultural Research Center, Beltsville, MD 20705 USA; Magnetic Resonance Laboratory “Annalaura Segre”, Institute of Chemical Methodologies, CNR, Monterotondo, Rome, Italy; Statistics Group, United States Department of Agriculture, Agricultural Research Service, The Henry A. Wallace Beltsville Agricultural Research Center, Beltsville, MD 20705 USA; Crop Systems Laboratory, United States Department of Agriculture, Agricultural Research Service, The Henry A. Wallace Beltsville Agricultural Research Center, Beltsville, MD 20705 USA; Department of Horticulture and Landscape Architecture, Purdue University, West Lafayette, IN 47907-2010 USA; University of Western Ontario, London, Canada

**Keywords:** Agro-environment and metabolomics, Ethylene, Metabolite networks, Methyl jasmonate, Polyamines, S-adenosylmethionine decarboxylase

## Abstract

**Introduction:**

Metabolomics provides a view of endogenous metabolic patterns not only during plant growth, development and senescence but also in response to genetic events, environment and disease. The effects of the field environment on plant hormone-specific metabolite profiles are largely unknown. Few studies have analyzed useful phenotypes generated by introducing single or multiple gene events alongside the non-engineered wild type control at field scale to determine the robustness of the genetic trait and its modulation in the metabolome as a function of specific agroecosystem environments.

**Objectives:**

We evaluated the influence of genetic background (high polyamine lines; low methyl jasmonate line; low ethylene line; and isogenic genotypes carrying double transgenic events) and environments (hairy vetch, rye, plastic black mulch and bare soil mulching systems) on the metabolomic profile of isogenic reverse genetic mutations and selected mulch based cropping systems in tomato fruit. Net photosynthesis and fruit yield were also determined.

**Methods:**

NMR spectroscopy was used for quantifying metabolites that are central to primary metabolism. We analyzed both the first moment (means) of metabolic response to genotypes and agroecosystems by traditional univariate/multivariate methods, and the second moment (covariances) of responses by creating networks that depicted changes in correlations of paired metabolites. This particular approach is novel and was necessary because our experimental material yielded highly variable metabolic responses that could not be easily understood using the traditional analytical approaches for first moment statistics.

**Results:**

High endogenous spermidine and spermine content exhibited strong effects on amino acids, Krebs cycle intermediates and energy molecules (ADP + ATP) in ripening fruits of plants grown under different agroecosystem environments. The metabolic response to high polyamine genotypes was similar to the response to hairy vetch cover crop mulch; supported by the pattern of changes in correlation between metabolites. Changes in primary metabolites of genotypes mutated for the deficiency of ethylene or methyl jasmonate were unique under all growth conditions and opposite of high polyamine genotype results. The high polyamine trait was found to dominate the low ethylene and low jasmonate mutations under field conditions. For several metabolites low ethylene and low methyl jasmonate genotypes had an inverse relationship. Collectively, these results affirm that interactions between metabolite pathways and growth environments are affected by genotype, and influence the metabolite quality of a crop.

**Conclusion:**

This study portrays how metabolite relationships change, both in mean and in correlation, under different genotypic and environmental conditions. Although these networks are surprisingly dynamic, we also find examples of selectively conserved associations.

**Electronic supplementary material:**

The online version of this article (doi:10.1007/s11306-016-1037-2) contains supplementary material, which is available to authorized users.

## Introduction

Metabolomics provides a view of endogenous metabolic patterns not only during plant growth, development and senescence but also in response to genetic events, biodiversity, environment and disease. Thus, to mention a few examples, metabolomics has contributed to mapping plant cell types (Moussaieff et al. 2013), modeling plant metabolic fluxes (Ratcliffe and Shachar-Hill 2006; Colombie et al. 2015), hormonal regulation of cellular metabolism (Carrari et al. 2006; Mattoo et al. 2006; Kausch et al. 2012; Lee et al. 2012; Aizat et al. 2014; Sobolev et al. 2014), and studies linking food composition to human (Hall et al. 2008) and veterinary health (Zicker et al. 2006). The application of the ‘omics’ technologies, including transcriptomics and metabolomics, is generating novel information on regulatory steps in metabolic pathways and how these metabolic networks behave during growth and development in plants (Carrari et al. 2006; Centeno et al. 2011; Brady et al. 2013; Moussaieff et al. 2013; Aizat et al. 2014).

Transgenic technology has resulted in unique crops, with more in the making (Cohen et al. 1973; Mattoo 2014). Transgenic research has enabled unambiguous confirmation of gene function in plants and characterization of novel genotypes that produce global changes in macro- and micro-molecules. Previously, we developed transgenic tomatoes homozygous for the genetic traits, fruit texture and metabolic/nutrient activity of tomato fruit, that modulate fruit shelf-life (Mehta et al. 2002; Kausch et al. 2012; Sobolev et al. 2014). One genotype was engineered with the yeast S-adenosylmethionine decarboxylase (SAMDC) gene under the ripening-specific E8 promoter, accumulating the polyamines spermidine (SPD) and spermine (SPM) in a fruit ripening-specific manner (Mehta et al. 2002). This genotype produces fruit which have higher processing quality, longer vine life and higher lycopene content (Mehta et al. 2002). A second genotype was developed with antisense-RNA to 1-aminocyclopropane-1-carboxylate synthase 2 (ACS2) gene under the CaMV 35S promoter. It constitutively suppresses the production of ripening hormone ethylene, producing half the ethylene of the control line, and has considerably longer shelf-life (Sobolev et al. 2014). Both polyamines and ethylene impact plant responses to abiotic stresses (Capell et al. 2004; Cramer et al. 2011). A third genotype was developed with co-suppression of the tomato lipoxygenase gene under the constitutive CaMV 35S promoter (Kausch et al. 2012). It is deficient in the stress hormone methyl jasmonate (meJAS) and has better texture than the control line (Kausch et al. 2012). Jasmonates have been recently implicated in temporal inhibition of ethylene biosynthesis and prevention of stamen desiccation during floral development in tomato (Dobritzsch et al. 2015).

We have described changes in the primary fruit metabolome of these tomato lines grown in a temperature and light controlled greenhouse (Mattoo et al. 2006; Kausch et al. 2012; Sobolev et al. 2014). Significantly up-regulated accumulation of amino acids such as glutamine (GLN), aspartate (ASP), threonine (THR), asparagine (ASN), glutamate (GLU), γ-aminobutyrate (GABA), and histidine (HIS), of ATP + AMP, and Krebs cycle intermediates, citrate and fumarate, in both high polyamine lines was observed (Mattoo et al. 2006). As expected, metabolomes of ethylene- and methyl jasmonate-deficient fruits were noticeably different from that of high polyamine fruit. Ethylene deficiency resulted in decreasing levels of most amino acids except ASP and GLU compared to the control line, and had higher PHE levels compared to high polyamine fruits. Methyl jasmonate-deficient fruits were remarkably different from high polyamine fruits when grown in the greenhouse, with levels of many amino acids decreased (Mattoo et al. 2006; Kausch et al. 2012).

The effects of the field environment on plant hormone-specific metabolite profiles are largely unknown. Few studies have analyzed useful phenotypes generated by introduction of single or multiple gene events alongside the non-engineered wild type control at field scale to determine the robustness of the genetic trait and modulation in the metabolome as a function of specific agroecosystem environments (Neelam et al. 2008; Kogel et al. 2010; Mattoo and Teasdale 2010). Sustainable agroecosystem management practices that protect the environment, minimize chemical inputs, and lower the costs of production while meeting the demand of a growing world population have become a priority in the past two decades. One such sustainable farming practice for tomato production is a reduced-tillage, cover crop based system that maximizes production while enhancing environmental services (Lu et al. 2000; Abdul-Baki and Teasdale 2007). The use of leguminous and non-leguminous cover crops grown prior to planting the cash crop in combination with the absence of tillage creates an agroecosystem which positively impacts soil microbial community structure, other soil environmental parameters, and crop performance in many traditionally bred crop plants including tomatoes (Abdul-Baki et al. 1996; Buyer et al. 2010). Growth of tomato plants on leguminous hairy vetch versus conventional black polyethylene mulch revealed differential regulation of select signaling pathways and likely crosstalk among plant organs affecting longevity and disease tolerance (Kumar et al. 2004; Neelam et al. 2008; Mattoo and Teasdale 2010).

Given this profound influence of the agroecosystem environment on the gene expression and physiology of traditionally bred tomato cultivars, we sought to determine the metabolomic profile of our transgenic tomato lines grown under a range of field growing conditions from conventional tillage and surface mulching with synthetic polyethylene sheets to the sustainable alternative system without tillage and mulching with residue of cover crop vegetation. We used NMR spectroscopy for quantifying metabolites, focusing on those that are central to primary metabolism. We analyzed both the first moment (means) of metabolic response to genotypes and agroecosystems by traditional univariate/multivariate methods, as well as the second moment (covariances) of response by creating networks that depicting changes in correlations of paired metabolites.

Previous research investigating metabolomic patterns have focused on the first (statistical) moment, i.e. changes in mean values, whereby observed metabolic mean values and differences between mean values are presented to describe the metabolomic state or change in state of the organism under study in response to an exogenous or genetic manipulation. We also investigated changes in the second (statistical) moment, covariances of metabolites; how the covariances responded to experimental treatment (Fukushima et al. 2011). Specifically, we characterized changes in the correlations between metabolite pairs among experimental treatments using both formal statistics and graphics for visualizing second moment changes in networks. These changes in the network of metabolites that occur with changing genetic/experimental conditions are implicitly assumed to be absent in traditional univariate and multivariate techniques. This approach is novel and was necessary because our experimental material, derived from field grown transgenic germplasm grown under contrasting agro-environmental conditions, resulted in highly variable metabolic responses that could not be easily understood using the traditional analytical approaches that look only at first moment statistics. We show here that the network defined by metabolite relationships in the tomato fruit metabolome are hormone and environment specific, demonstrating the highly plastic nature of the fruit primary metabolome.

## Materials and methods

### Field operations and experimental design

Field trials were conducted on the North Farm of Beltsville Agricultural Research Center, Beltsville, Maryland, USA, in 2006 and 2007. Field operations were conducted in areas adjacent to those used for tomato experiments as previously described (Buyer et al. 2010) and were conducted according to similar methods and timings as were used in those experiments. In contrast to (Buyer et al. 2010), however, only four mulching system management treatments were employed in these experiments, (1) black polyethylene (BP) mulch laid on beds prepared by spring tillage, (2) hairy vetch (HV) cover crop mulch on untilled beds, (3) rye (RY) cover crop mulch on untilled beds, and (4) untilled beds with no cover crop mulch, hereafter designated as bare soil (BS) (included in the 2007 experiment only). In the September before the tomato cropping season, lime and nutrients other than nitrogen were applied to fields according to Maryland soil test-based recommendations and beds, 1.5 m center to center, were formed. In late September, hairy vetch was planted at 45 kg ha^−1^ and rye was planted at 101 kg ha^−1^ on the surface of designated beds. The beds to receive black polyethylene or to remain bare soil were maintained free of vegetation by applying 0.6 kg ha^−1^ of paraquat in late October and again in early spring. The black polyethylene mulch treatment was prepared by rototilling the designated bed area, shaping a new bed, installing drip irrigation lines 5 cm deep within the bed, and laying 1.2 m wide black polyethylene sheets over the surface of the beds. HV and RY cover crops were mowed such that residue was dropped in place to leave a mulch on the surface of the beds (Abdul-Baki and Teasdale 2007). The beds were left undisturbed with no surface mulch in the bare soil treatment. Drip irrigation lines were laid on top of the mulch and soil in the undisturbed HV, RY, and bare soil treatments.

Tomato genotypes were transplanted along the center of beds in early June. The experiment was designed as a split-plot design with mulch treatment as the whole plot and genotype as the subplot. Five plants of each genotype were planted in each mulch treatment in each of four replications with 56 cm between plants. A spacing of 112 cm was left between each genotype along the bed. Beds were irrigated at least weekly using drip irrigation. Nitrogen was applied through the drip irrigation system at four times approximately corresponding to transplant establishment, flowering, fruit initiation, and initial fruit ripening stages of development. A total of 224 kg ha^−1^ of N was applied to all treatments without hairy vetch and a total of 112 kg ha^−1^ of N was applied to the hairy vetch treatment based on previous research that defined the requirements for achieving optimum yields (Abdul-Baki and Teasdale 2007).

### Genotypes and construction of transgenic lines

Transgenic tomatoes (in Ohio 8245 background) homozygous for the indicated genetic event(s) were tested alongside the azygous control line (556AZ): Five genotypes (556HO, 579HO, 650-12HO, LS-4 HO and 102AS-1HO) in 2006, and an additional line 2AS-2HO was added to the field trial in 2007. The codes used for each tomato line (genotypes) are summarized in Supplementary Table 1. These included three types of engineered genotypes of tomato with the modulated fruit shelf-life, fruit texture and metabolic/nutrient activity of tomato fruit. Thus, individual genetic events included tomato lines engineered with yeast SAM decarboxylase gene under ripening-specific E8 promoter (*Spe2*, accession # M38434; ySAMdc) in a fruit specific manner for enhanced metabolic activity and accumulate polyamines spermidine and spermine in a fruit ripening-specific manner (556HO and 579HO, lines 8 and 10 (Supplementary Table 1), which have higher processing quality, longer vine life and higher lycopene content (Mehta et al. 2002); antisense-RNA to 1-aminocyclopropane-1-carboxylate synthase 2 (*ACS2*) gene under CaMV 35S promoter to constitutively suppress the production of ripening hormone ethylene (2AS-2HO, line 2), which produces half the ethylene than the control 556AZ line and has considerably longer shelf-life (Sobolev et al. 2014); co-suppression of tomato lipoxygenase gene under the constitutive CaMV 35S promoter [650-12HO, line 12, which is deficient in the stress hormone methyl jasmonate (meJAS) and has better texture than the control line (LOX; *SlLoxB,* accession # U13681) (Kausch et al. 2012). Additional tomato lines were developed with double genetic events by making backcrosses between one high polyamine line (line 10) and ethylene-suppressed line 2 (102AS-1HO, line 4), as well as another high polyamine line (line 8) and meJAS-deficient line 12 (LS-4HO, line 20) with an azygous line as a control (description in Supplementary Table 1). These lines offered a resource to study interactions between polyamines and indicated hormones in a unique germplasm and determining differences when grown in fields with different agroecosystem environments.

### Whole plant measurements

Single leaf CO_2_ and H_2_O vapor exchange measurements were made on clear days in 2006 and 2007 using a CIRAS-1 portable photosynthesis system (PP Systems, Amesbury, MA) under ambient mid-day conditions of light, temperature, humidity and CO_2_ concentration, using a broad-leaf cuvette. Measurements were made on July 18, Aug 01, Aug 17 and Sep 07 in 2006 and on Aug 02, Aug 14, Aug 27, and Sep 12 in 2007. Leaf temperatures ranged from 26 to 30 °C across all measurement dates. Mean values of the leaf to air difference in water vapor pressure ranged from 1.2 to 1.8 kPa for the different measurement dates. Photosynthetic photon flux density exceeded 1200 µmol m^−2^ s^−1^ for all measurements, and the CO_2_ concentration external to the leaves was controlled at 370 µmol mol^−1^. Leaves selected for measurement were recently fully expanded upper canopy leaves fully exposed to light, and were found to have the highest rates of any leaves on the plants. On each date, gas exchange was measured on one leaf of each genotype in each replicate plot. The order of measurements of genotypes within mulch treatments was random, and the mulch treatments were measured such that the mean time of day was equalized. Measurements were completed in about 2 h on each date.

### Determination of fruit yield

In 2006, pink and red fruit (the range of fruit developmental stage normally harvested commercially) were harvested from the five-plant subplots from each replication on two dates, the first harvest took place on Sep 12, and the final harvest on Sep 22. In 2007, the five-plant subplots were harvested from three replicates at two times. For the first harvest, all the pink and red fruits were harvested Sep 6 to 9. The same plants were harvested for a second time on Sep 25 to 28. The number and weight of fruit per plant and average fruit weight were calculated both for the early harvest and for the total of the two harvests.

### Metabolite analysis

The levels of biogenic amines—putrescine, spermidine and spermine—in pericarps of fruit from transgenic and azygous lines grown on HV, BP, RY and Bare were determined as described previously (Minocha et al. 1990). Pericarp tissue was powdered in liquid nitrogen and lyophilized, and 100 mg (dry weight) of each sample was mixed with 800 μl of 5 % perchloric acid and stored at −20 °C. The frozen samples were thawed at room temperature and the freeze and thaw cycles repeated three times. Finally, the samples were centrifuged at 12,000×*g* for 15 min and the supernatants assayed (Minocha et al. 1990; Mehta et al. 2002).

NMR spectroscopy was employed to profile 30 metabolites classified into simple sugars, organic acids, amino acids, energy molecules, choline and nucleic acid related. The names and their abbreviations used in the text are listed in Supplementary Table 2. Prior to analysis, pericarp tissues from pink and red fruit of all transgenic genotypes and non-transgenic azygous line collected from field grown plants were chilled in liquid nitrogen immediately after slicing and stored at −80 °C. The frozen tissue was powdered in liquid nitrogen and lyophilized. Samples of the dry powder were then analyzed by NMR spectrometry as previously described (Sobolev et al. 2003; Mattoo et al. 2006).

### Quantitative PCR analysis

Fruits were harvested from the genotypes 556HO, 579HO, 650-12HO and LS-4 HO. Total RNA was isolated from lyophilized pericarp samples using the RNeasy Plant Mini Kit (Qiagen). RNA quantification and quality were determined using a spectrophotometer and native agarose gel electrophoresis, respectively. First strand cDNA synthesis was performed using the SuperScript^®^ cDNA Synthesis Kit (Invitrogen) following the manufacturer’s instructions, in a final volume of 20 μl. The final cDNA products were diluted tenfold prior to use in real-time RT. Quantitative RT-PCR (Q-PCR) was performed with gene-specific primers (Supplementary Table 3), which were designed using Primer3 software. The Q-PCR conditions were the same as before (Mattoo et al. 2006; Fatima et al. 2012). Tomato actin gene was used as reference for normalization and transcripts were quantified in BP and HV treated samples by the comparative C_T_ method using the $2^{{ - \varDelta \varDelta {\text{C}}_{\text{T}} }}$ formula as described (Livak and Schmittgen 2001). Gene transcripts of E8, S-adenosylmethionine decarboxylase (SAMDC), lipoxygenase (LOX) were quantified.

### Statistical analyses

Univariate analyses were performed with analysis of variance (ANOVA) using the mixed model procedure of SAS version 9.2 (SAS Institute, Cary, NC). The model for analyzing fruit yield variables, photosynthesis variables, polyamines and metabolites had mulch, genotype and mulch by genotype interaction treatments as fixed effects. Year and field block nested within year were random effects for the fruit yield analyses, year and block within year and sampling date within year were random effects for the photosynthesis analyses, and year and fruit stage were random effects for the polyamine and metabolite analyses. Principal components analysis (PCA) was performed on the metabolite data set after standardization to mean zero and standard deviation one. Two ANOVA’s were performed, one for each of the first two components using mulch, genotype and mulch by genotype interaction treatments as fixed effects.

Metabolites were organized into groupings of amino acids, organic acids, sugars, and energy metabolites in order to facilitate analysis of overall trends within groups. Each metabolite was standardized to mean = 0 and standard deviation = 1 to give equal weighting to each metabolite. An ANOVA was then performed on the standardized metabolic data by group with genotype or mulch treatment as a fixed effect.

Canonical correlation analysis (CCA) was performed to explore multivariate correlations between whole plant variables (early fruit number, early fruit weight, early weight per fruit, total fruit number, total fruit weight, total weight per fruit, net photosynthesis, stomatal conductance, and internal CO_2_ concentration) and metabolite variables. Univariate Pearson correlations between these variables were also calculated.

A single network analysis for metabolites (Langfelder and Horvath 2008; DiLeo et al. 2011), assuming an unvarying correlation structure among variables over the whole data set, was found not adequate to describe our metabolite networks and their dynamics under the genotype and mulching conditions we observed. We introduce a new approach based on characterizing changes in simple correlations between metabolite pairs among subsets of data. We partitioned the data into subsets of interest, e.g., genotypes representing low versus high polyamine plants, in order to obtain sufficient sample sizes for calculating correlations. This approach is analogous to an ANOVA with only crossed main effects (indeed, few interactions were found in univariate analyses), where statistical testing of main effects is done averaging over other data partitions.

To determine which changes in correlations were the important ones to depict in our networks, we established a confidence interval for the hypothesis of no change in correlation (*P* < 0.05, two-tailed) for individual metabolite pairs, following the Fisher *r*-to-*z* transformation, and show only large changes in correlation (for *P* < 0.05). Pearson correlations were calculated using the R statistical software (R Core Team 2014), and tests for significant differences between correlations using the psych package (Revelle 2014). Data sets with the information necessary for visualizing changes in metabolic networks in Cytoscape (v. 3.1.1, Shannon et al. 2003) were exported as csv files (these included the metabolite names and *z* value differences). We did not include correlation changes involving the uncharacterized “B” metabolite. We color-coded the networks, with blue representing increases in correlation (a smaller positive correlation becoming a larger positive correlation) and red representing decreases in correlation (a larger positive correlation becoming a smaller positive correlation). The thickness of the lines joining the metabolites was used to represent the magnitude of the change (on the *z* scale), with the thinnest line representing a change of about 0.1 in correlation and the thickest a change of about 0.7 in correlation. An important point to remember when looking at the figures is that they do not represent the correlations themselves, rather they represent changes in correlation. Thus, two metabolites that are highly correlated in both subsets of data would not be linked in the figure because their correlation did not change.

We are aware that analyses repeated in different ways on the same data set leads to correlated test statistics. The *P* values can be adjusted for this accurately if the nature of the correlation among tests is known (not true for our case) or more generally, using approaches such as false discovery rate (Benjamini and Hochberg 1995). Since the interests in this paper are primarily exploratory, we have retained the traditional *P* < 0.05 standard for significance throughout, accepting that this is liberal and does not control the experiment-wise error rate (still an active research area in statistics).

## Results

### Confirmation of system function under field grown conditions

We first ascertained the stability of the introduced genes in tomato plants in the field and/or their biochemical phenotype. In addition to the engineered lines mentioned above, we developed and tested additional tomato lines with double genetic events by making backcrosses between one high polyamine line (line 10) and ethylene-suppressed line 2 (102AS-1HO, line 4), as well as another high polyamine line (line 8) and meJAS-deficient line 12 (LS-4HO, line 20) with an azygous line as a control (description in Supplementary Table 1). Q-PCR data of three genes—yeast SAM decarboxylase (*ySAMDC*), tomato E8 (*SlE8*) and tomato lipoxygenase (*SlLOX*) in different tomato genotypes grown in BP (black plastic mulch) and HV (hairy vetch mulch) are shown in Supplementary Fig. 1. As expected, lines 8 and 10, carrying the *ySAMDC* transgene, expressed *ySAMDC* transcripts. However, line 20 had higher expression of *ySAMDC* transcripts than its parent line 8. Specific effects of the BP versus HV were evident: *ySAMDC* transcript expression in line 20 (G20) was several-fold enhanced under BP (Supplementary Fig. 1, ySAMDC). To ascertain if this BP effect was due to an effect on the *SlE8* promoter used to drive the expression of the chimeric *ySAMDC* construct, the accumulation of *SlE8* transcripts was also quantified under BP and HV. The pattern of *SlE8* transcript accumulation in BP-grown lines 8 and 10 was similar to the pattern of *ySAMDC* transcripts (Supplemental Fig. 1, compare E8 with ySAMDC under BP). In fact, BP-grown line 12 had higher expression *of SlE8* transcripts than when grown under HV (Supplementary Fig. 1, E8), and in contrast these transcripts in control azygous line 5 accumulated maximally under HV. *SlLOX* transcripts were suppressed in both line 12 and its sibling, genotype 20, as expected while mulch systems had little differential effect. HV mulch had a more enhancing effect on *SlLOX* expression in line 5 (G5) and line 8 (G8). In general, the relative accumulation levels of *SlE8* transcripts were considerably lower than *ySAMDC* and *SlLOX* transcripts.

We introduced SAMDC into tomato (Mehta et al. 2002) because we anticipated that among the main polyamines present in tomato—putrescine (Put), spermidine (Spd) and spermine (Spm)—Spd and Spm should accumulate and Put should decline in lines 8 and 10, and their siblings 4 and 20. The values for the three polyamines (Supplementary Table 4) showed the expected pattern of polyamines for the indicated genotypes. The siblings (lines 4 and 20) behaved like their high polyamine parents qualitatively and quantitatively, with line 20 having elevated spermine levels compared to all other tested genotypes and consistent with the elevated SAMDC expression in this genotype (Supplementary Fig. 1). In terms of polyamine content, the methyl jasmonate (meJAS) deficient (line 12) and ethylene deficient (line 2) genotypes behaved similar to the azygous control line 5. This analysis also indicated no significant mulching system main effects or genotype*mulch interactions in the pattern of polyamine accumulation.

### Net photosynthesis and fruit yield as a function of genotypes and mulching system

Constitutive suppression of ethylene was found to result in higher net photosynthesis and stomatal conductance in line 2 than in the azygous control (Supplementary Table 5). This was true also for its sibling line 4 (crossed with high polyamine line 10), which behaved like parent 2 for all variables. In contrast, line 10 with fruit-specific high polyamine content had the lowest net photosynthesis, stomatal conductance and fruit yield. This phenotypic trait was, as expected, absent from line 4, likely because of the differential regulation of the genetic events introduced, suggesting that this fruit-specific event was not transferred to vegetative parts of the tomato plant. In all genotypes, the differences in net photosynthesis were found unrelated to internal CO_2_ levels (Supplementary Table 5). There were no significant effects for any of these variables among genotypes 5, 8, 12, and 20.

**Fig. 1:**
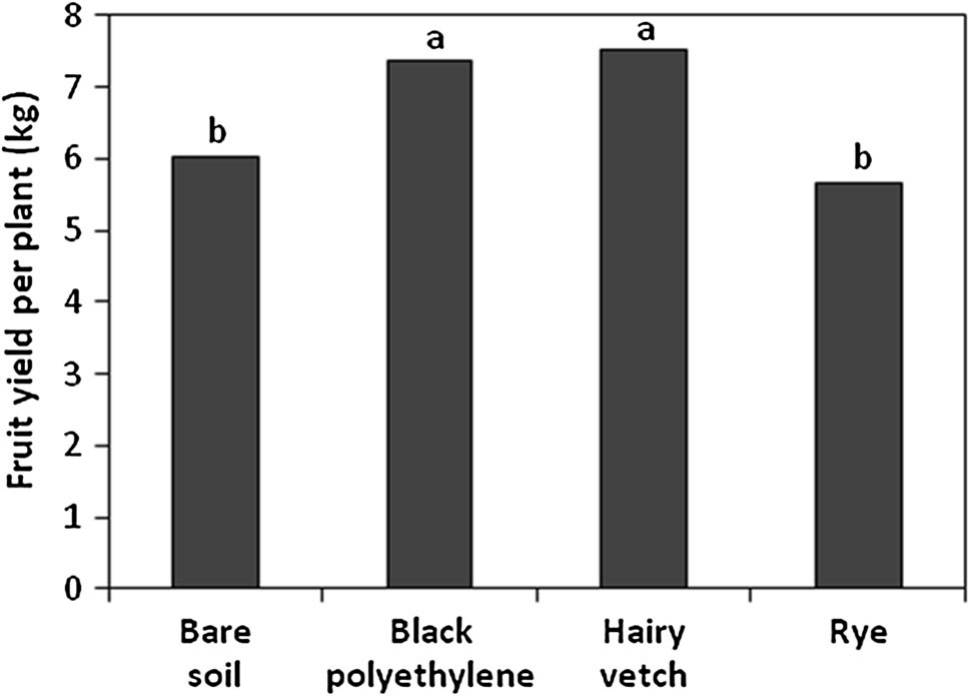
Effect of mulch treatments on tomato fruit yield. Least square means of 2006 and 2007 growing seasons. *Bars* with the *same letter* are not significantly different (*P* < 0.05)

Tomato fruit yield was higher in plants grown in hairy vetch or black polyethylene mulch than in rye mulch or bare soil (Fig. 1), in line with previous reports (Buyer et al. 2010). However, there were no significant effects of mulching system on photosynthetic variables.

### Principal components analysis of metabolites

**Fig. 2:**
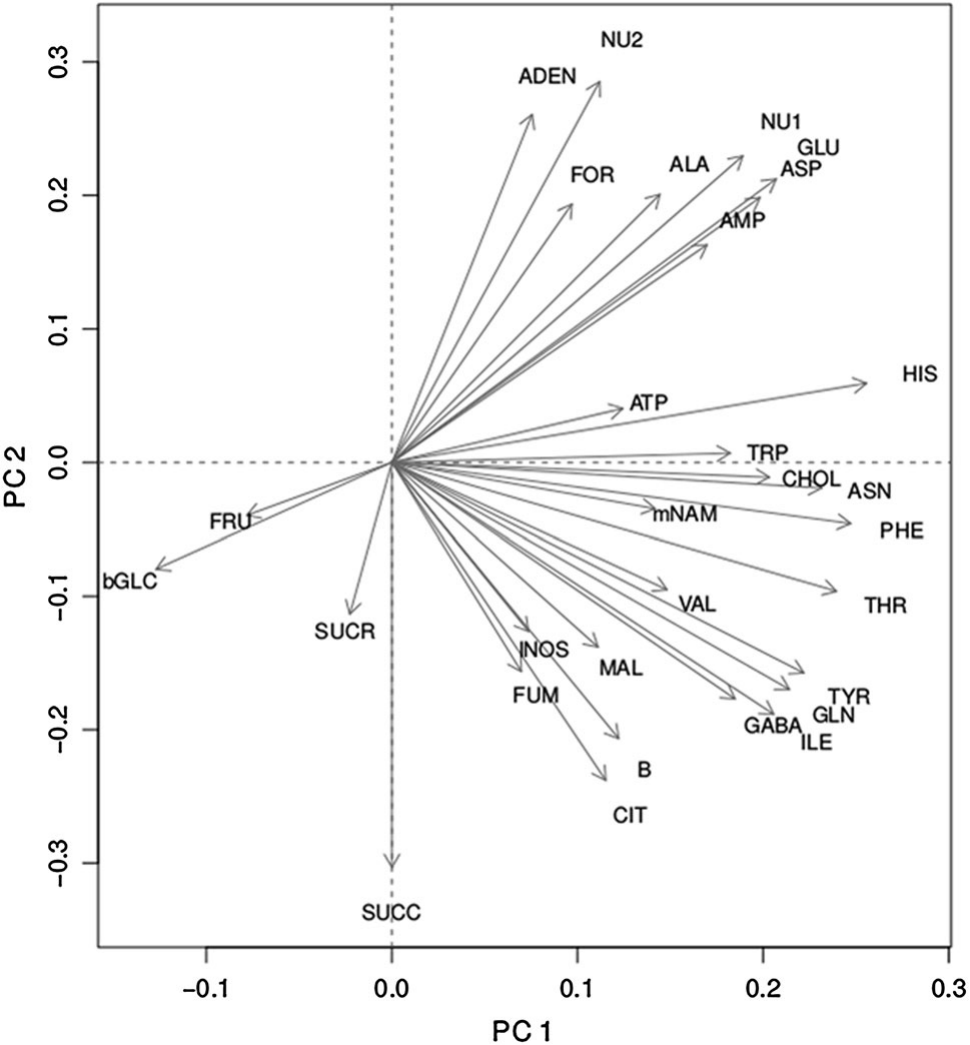
Eigenvectors of metabolite variables plotted on the first two axes of a principal components decomposition. Variables were first standardized to mean zero and standard deviation one. *Dotted lines* delineate the four quadrants

Principal components analysis (PCA) was performed on the metabolite data set after standardization to zero mean and unit standard deviation. PCA decomposed the metabolome data set to two principal components with 36 % of the explainable variation described by the first component and 20 % of the explainable variation described by the second component. Other components each described less than 10 % of this variation. The first principal components axis was most positively correlated with the metabolites histidine (HIS), asparagine (ASN), phenylalanine (PHE), and threonine (THR) (eigenvectors > +0.25) and most negatively correlated with glucose (bGLC) and fructose (FRU) (eigenvectors < −0.08) (Fig. 2). The second principal components axes was most positively correlated with the metabolites adenosine (ADEN), NU1, and NU2 (eigenvectors < +0.25) and negatively with citrate (CIT), and succinate (SUCC) (eigenvectors > −0.25) (Fig. 2). Our PCA analysis was followed by an ANOVA on the first two components as dependent variables. We used mulching system and genotype as the independent variables (with and without interaction) and found that none of these independent variables were significant (all *P* values >0.05, results not shown). Although this analysis did not adequately resolve genotype and mulch system treatments, we further explored the data set, looking at the variables independently using univariate ANOVA, and then with metabolite networks.

### Univariate ANOVA of metabolite profiles

The univariate ANOVA for each metabolite separately showed several significant genotype and mulching system main effects, and very few significant interactions between these effects. The lack of significant mulch*genotype interactions implies that the pattern of metabolite responses to mulching system are consistent across genotypes and that the pattern of genotype responses are consistent across mulches. Therefore, we report on the main effects.

The high polyamine genotypes 8 and 10 had generally higher levels of the amino acids alanine and asparagine, organic acids citrate and succinate, and ATP + ADP levels than the meJAS deficient line 12 and the ethylene deficient line 2 (Supplementary Table 6). In contrast genotype 2 had higher levels of inositol and adenosine than either genotypes 8 and 10. Genotype 10 had higher levels of choline and mNAM than all other genotypes. Sibling 20 was more similar to its parent genotype 8 for valine, citrate, succinate, and adenosine, but more similar to genotype 12 for asparagine. Sibling 4 was more like its parent genotype 10 for valine, inositol, and adenosine, but more like its parent genotype 2 for choline and mNAM.

Significant univariate effects due to agroecosystem environment, created by different mulches, on amino acids, organic acids, sugars, and energy fruit metabolites are summarized in Supplementary Table 7. The hairy vetch (HV) mulching system led to higher levels of selected amino acids (glutamate, threonine, GABA), and organic acids (citrate, malate, succinate) and mNAM, and lower levels of the sugars (glucose and fructose). Like HV, rye mulch (Rye) also increased glutamate and threonine but its effect on aspartate was greater than HV, the black polyethylene (BP) mulching system, and bare soil (BS) (Supplemental Table 2). In contrast to HV and Rye systems, both glucose and fructose, and NUC2, were higher under BP and BS environments.

**Fig. 3:**
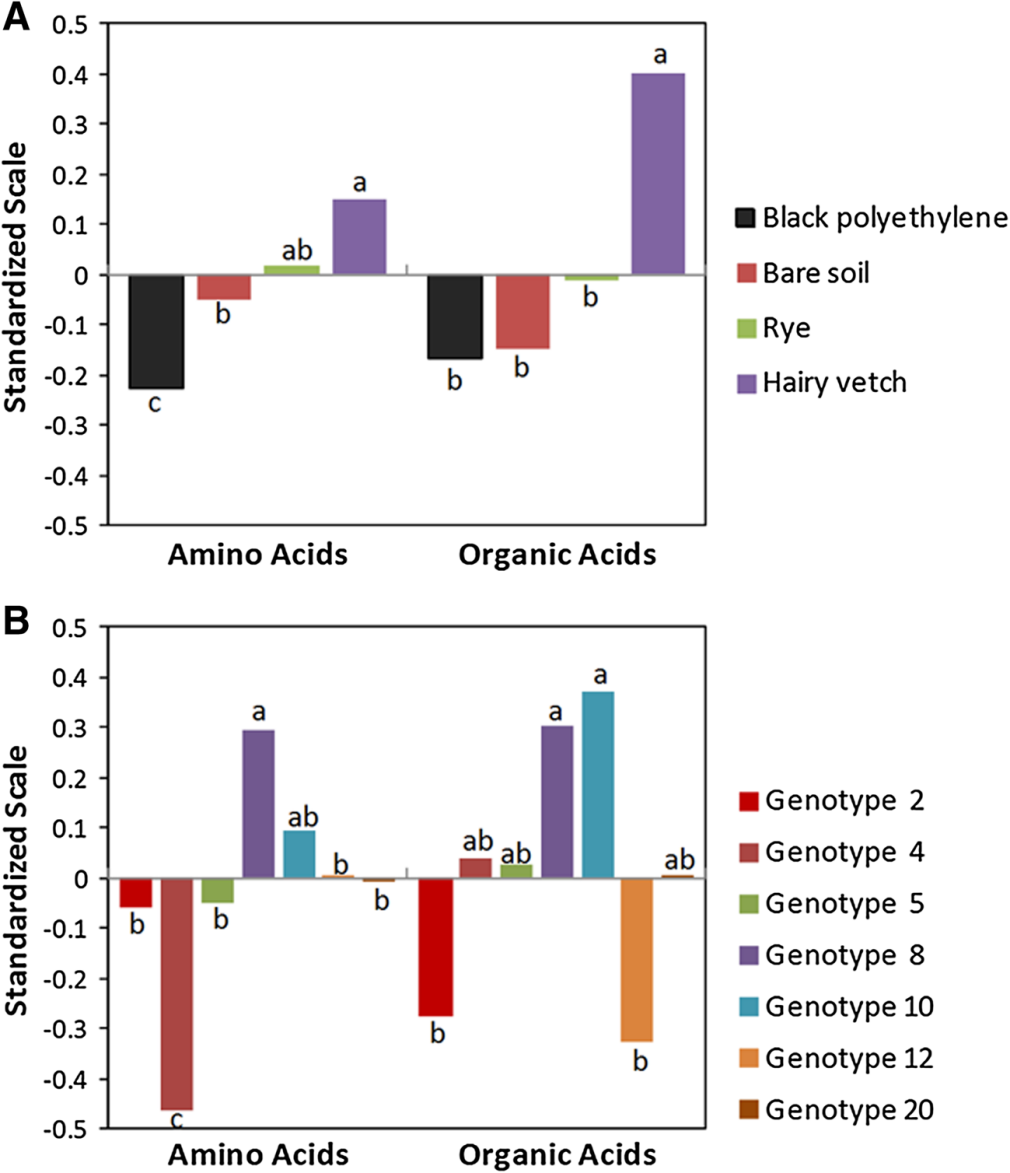
Amino acid and organic acid response to mulch treatment (**A**) or genotype (**B**).* Bars* are the mean of all amino acids or organic acids analyzed in this experiment after data for each metabolite was standardized to mean = 0 and standard deviation = 1. *Bars* within each metabolite group followed by the same letter are not significantly different (*P* < 0.05). Genotype codes are defined in Supplementary Table 1

The univariate analyses suggested that there were common metabolic patterns of response for selected genotypes and mulching systems, particularly among amino acids and organic acids. Consequently, each metabolite was standardized to mean zero and unit standard deviation and analyzed by ANOVA across all metabolites for each metabolic grouping. There were significant differences (*P* < 0.05) among standardized amino acid and organic acid means for both mulch and genotype, but not for means of standardized sugar or energy metabolites (Supplementary Tables 6 and 7). Plants grown in the HV mulch system had higher overall amino acid and organic acid levels, whereas plants grown in the BP mulch system had the lowest levels (Fig. 3). The high polyamine genotypes 8 and 10 also had the highest overall amino acid and organic acid levels, whereas the ethylene-deficient and/or meJAS-deficient genotypes had the lowest levels (Fig. 3). A summary of this analysis and the univariate analyses showed that agroecosystem environment created by HV mulch system induced a metabolic profile similar to that induced by high polyamine genotypes (lines 8 and 10).

### Correlation analysis of whole plant versus metabolite data sets

A number of plant photosynthesis/yield variables, in addition to the fruit metabolite variables, were collected. A dimension reduction technique, canonical correlation, was used to examine the multivariate relationship between plant and fruit metabolite variables, but was not significant (Wilks’ Lambda = 0.4658). Univariate correlations between plant and fruit metabolite variables were generally low (the average absolute value of all 297 correlations of the 9 plant by 33 metabolite variables was 0.213). Thus, there was little linkage between plant performance and fruit metabolism in these genotypes and mulching systems.

### Metabolic networks in tomato fruit change with genotype and ecosystem environment

When we used PCA and WCNA (Langfelder and Horvath 2008) for discerning the patterns of eigenmetabolites over treatments, we found that these approaches did not adequately capture how metabolites responded to genotype and treatment changes. The WCNA approach is based on the assumption that the correlation structure of the data does not vary across treatment effects, though means may be affected by treatments. Instead, we found that the observed relationships among the metabolite pairs often changed substantially across the different genotypes and mulching systems in our experiments. To discern how the networks of metabolites changed as conditions varied, we used an approach based on characterizing *changes* in correlations between metabolite pairs among subsets of data. A listing of the correlation between all pairs of metabolites for each data subset is presented in Supplementary Table 8. The data were partitioned into subsets of interest for this study, namely, (a) genotypes whose fruit have low (lines 2, 5 and 12) versus high polyamine content (Spd and Spm) (lines 4, 8, 10 and 20), (b) normal (lines 2, 4, 5, 8 and 10) versus deficient (lines 12 and 20) meJAS content, and (c) mulch system in which the plants were grown (two categories: bare and black polyethylene versus hairy vetch and rye cover mulches).

**Fig. 4:**
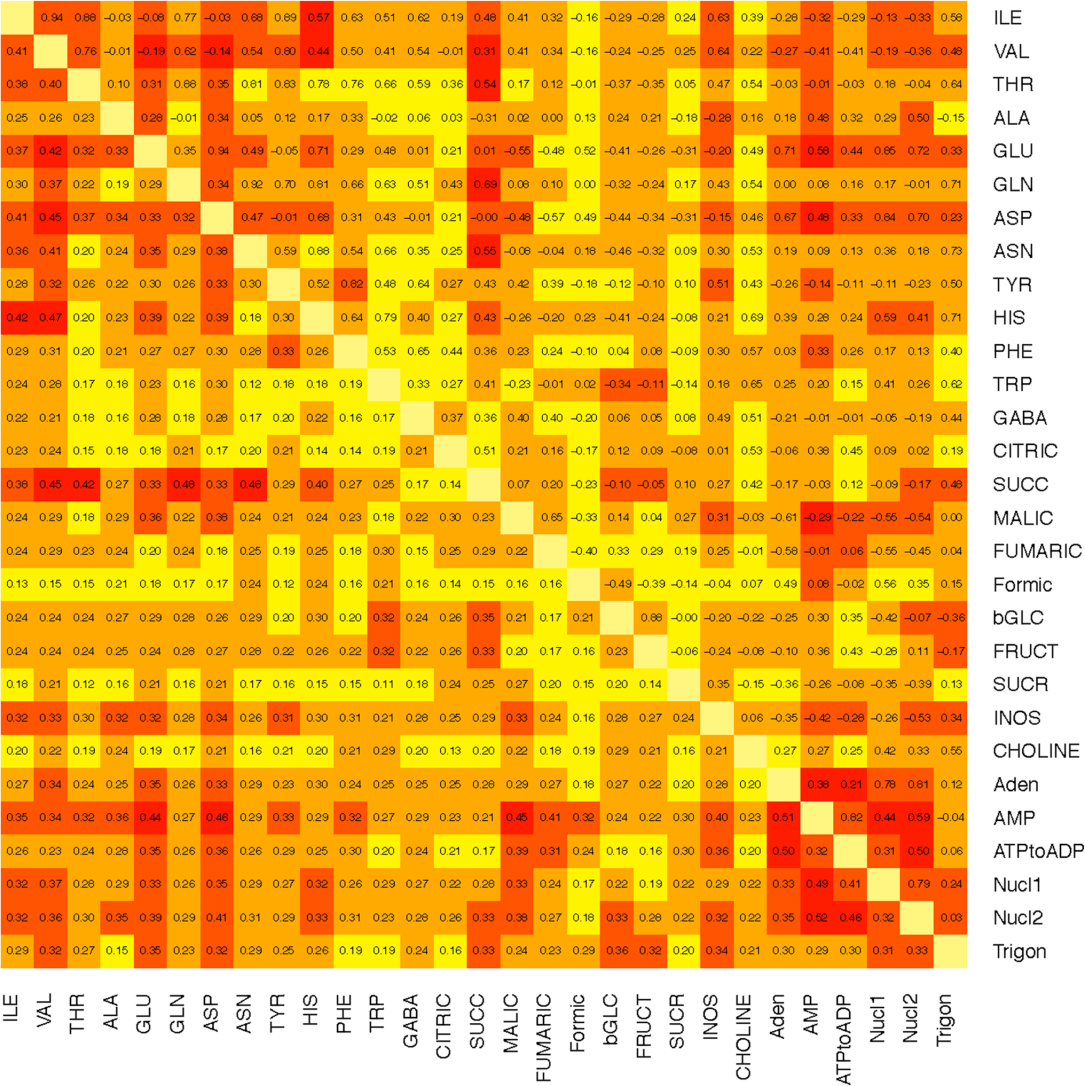
*Five color* heat map of stability of pairs of metabolites across all treatments, based on the variability (as standard deviations) of correlations, following Fisher’s *Z* transformation. The least stable pairs, those responding most to changes across treatments, are the darkest red, the most stable are *yellow*. The *upper right triangle* gives the average correlation of each pair, the *lower left triangle* gives the standard deviations used to create the heat map

Figure 4 summarizes data on the least and most stable metabolite pairs across all treatments based on the variability of correlations following Fisher’s *Z* transformation. Stability was calculated as the standard deviation of transformed correlations of metabolite pairs across all partitions of the data described above (e.g. one partition is high versus low polyamine). The least stable metabolite pairs are colored darkest red (mean standard deviation >0.35 on the *Z* scale) in the heat map while the most stable are yellow (mean standard deviation <0.19 on the *Z* scale). The most stable was the pair, TRP-SUCR, with the smallest standard deviation of 0.109, and the least stable was AMP-Nucl2, with the highest standard deviation of 0.518. Metabolites that had the most stable associations with other metabolites were citrate and formate (14 and 13 associations with standard deviations <0.19, respectively, primarily with amino acids and organic acids) and sucrose (12 associations <0.19, primarily with amino acids). Correlational relationships amongst these metabolite pairs with low standard deviations were least disturbed by perturbations caused by differences in genotype or mulching system and represent the most stable linkages between metabolites in this experiment. In contrast, metabolite pairs with large standard deviations illustrate pairs whose linkage changed most in contrasting treatments; analyzed in more detail below.

Cytoscape (v. 3.1.1) (Shannon et al. 2003) was used for creating figures that defined networks of metabolic correlations in the contrasting treatment subsets described above (Supplementary Figs. 2–4). In the low Spd and Spm polyamine fruit, lines 2, 5 and 12 (Supplementary Table 1), high correlations between metabolites are visually evident for nodes NU1, ILE, VAL, THR, GLU, GLN, GABA, CIT, CHOL, INOS and SUCC followed by TYR, HIS, PHE and TRP (Supplemental Fig. 2A). Correlations were even stronger for NU1, ILE, THR, GLU, GLN, GABA, CHOL and TYR, and lower for VAL, CIT, INOS, mNAM in the high Spd and Spm polyamine fruit (represented by lines 4, 8, 10 and 20) (Supplementary Fig. 2B). In addition, metabolites with high correlations in the high polyamine lines include ADEN, AMP, NU1, NU2, ASP, ASN, HIS, MAL and FOR (Supplemental Fig. 2B), suggesting that positive interactions between these metabolites were enhanced, some of these being unique. Many high correlations were found between metabolites in tomatoes with normal content of meJAS, though correlations tended to be lower for nodes of SUCR, FUM, TRP and mNAM (Supplementary Fig. 3A, B). In comparison, the tomato lines deficient in the content of meJAS had a somewhat different pattern of correlations, with a weakening of some correlations, such as the nodes represented by ADEN, mNAM, GLN, ASN, TYR, TRP, GABA, SUCC, MAL and FUM (Supplementary Fig. 2B).

**Fig. 5:**
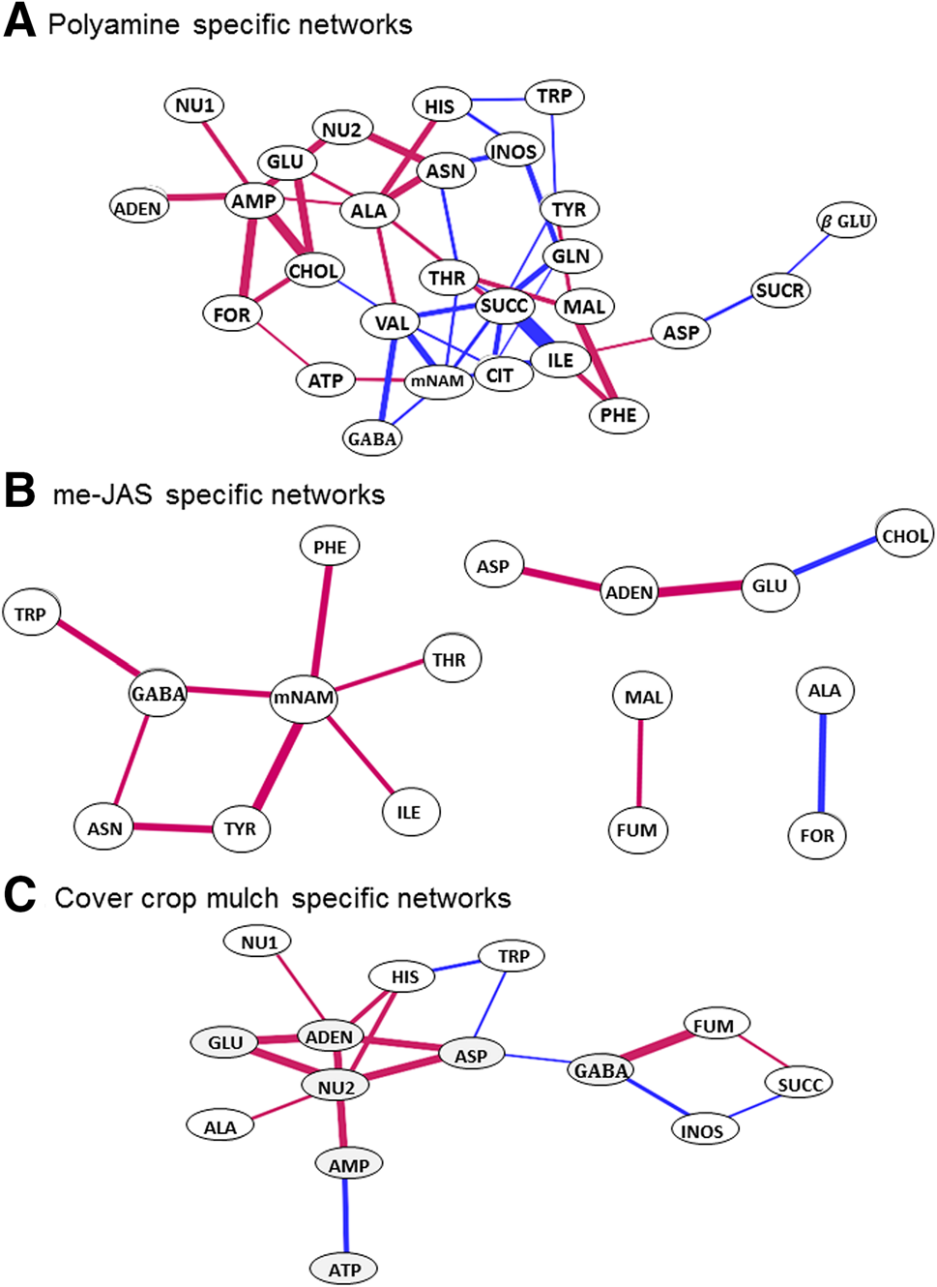
Changes in metabolic networks. (**a**) Polyamine-specific network changes, comparing metabolite pairs in low (genotypes 2, 5, 12) to high (genotypes 4, 8, 10, 20) polyamine content fruits (Supplementary Fig. 2); only significant changes (*P* < 0.05) in the correlations are shown. Decreases in correlation from low to high genotypes ranged from −0.688 to −0.214, shown in blue, the width of the line indicating the amount of decrease. Correlations that significantly increased from low to high polyamine genotypes, are shown in *red*, ranging from 0.614 to 0.122, the width of the line indicating the amount of increase. Mulch types were pooled for these calculations. (**b**) Methyl jasmonate (me-JAS)-specific network changes comparing metabolite pairs in low (genotypes 12, 20) to normal (genotypes 2, 4, 5, 8, 10) me-JAS fruits (Supplementary Fig. 3); only significant changes (*p* < 0.05) in the correlations are shown. Decreases in correlation from low to normal genotypes ranged from −0.432 to −0.476, shown in *red*; the width of the line indicating the amount of decrease. Correlations that significantly increased from low to normal me-JAS genotypes, are shown in *blue*, ranging from 0.217 to 0.461, the width of the line indicating the amount of increase. Mulch types were pooled for these calculations. (**c**) Cover crop mulch based changes in metabolite networks (Supplementary Fig. 4). Plants grown with mulches B (bare) and BP (black plastic) were compared to those grown with HV (hairy vetch) and RY (rye grass); only significant changes (*P* < 0.05) in the correlations are shown. Decreases in correlation from B, BP to HV, RY ranged from −0.242 to −0.416, shown in blue; the width of the line indicating the amount of decrease. Correlations that significantly increased from B, BP to HV, RY are shown in red, ranging from 0.232 to 0.534, the width of the line indicating the amount of increase. All genotypes were pooled for these calculations

Because it was difficult to discern differences between contrasting treatments in these complex correlation networks, the most significant *changes* in the correlation between metabolites shown in these supplementary figures were determined using a cut-off of *P* < 0.05 and aligned into networks using Cytoscape as shown in Fig. 5a–c. Blue colored lines represent significant strengthening in correlation (a smaller positive correlation becoming a larger positive correlation) and red represents significant weakening in correlation (a larger positive correlation becoming a smaller positive correlation). Again, note that these do not represent the correlations themselves, rather they represent changes in correlation between two contrasting treatment groupings.

The changes in metabolite networks, shown in Fig. 5a, depict significant changes in correlation between high polyamine and low polyamine lines, blue lines representing pairs of metabolites that become significantly more strongly coupled in high polyamine lines, while the red lines represent pairs of metabolites that are strongly coupled in high polyamine lines but become significantly less coupled in low polyamine lines. Many of the same metabolites that differed between high or low polyamine genotypes in the univariate analyses are also important nodes in the network exhibited in Fig. 5a. These metabolites clearly define the shift in metabolic activity in high versus low polyamine fruit.

Few significant changes in correlation between low and normal meJAS were observed as shown in Fig. 5b. Four unique and unlinked correlation networks were seen: MAL and FUM, ALA and FOR, ASP, ADEN, GLU and CHOL, along with a larger composite network. Most significant changes, going from low to normal meJAS, were a weakening of the correlations (red lines), only the GLU-CHOL and ALA-FOR pairs became more tightly coupled.

#### Metabolic network correlations as a function of agroecosystem environment

**Fig. 6:**
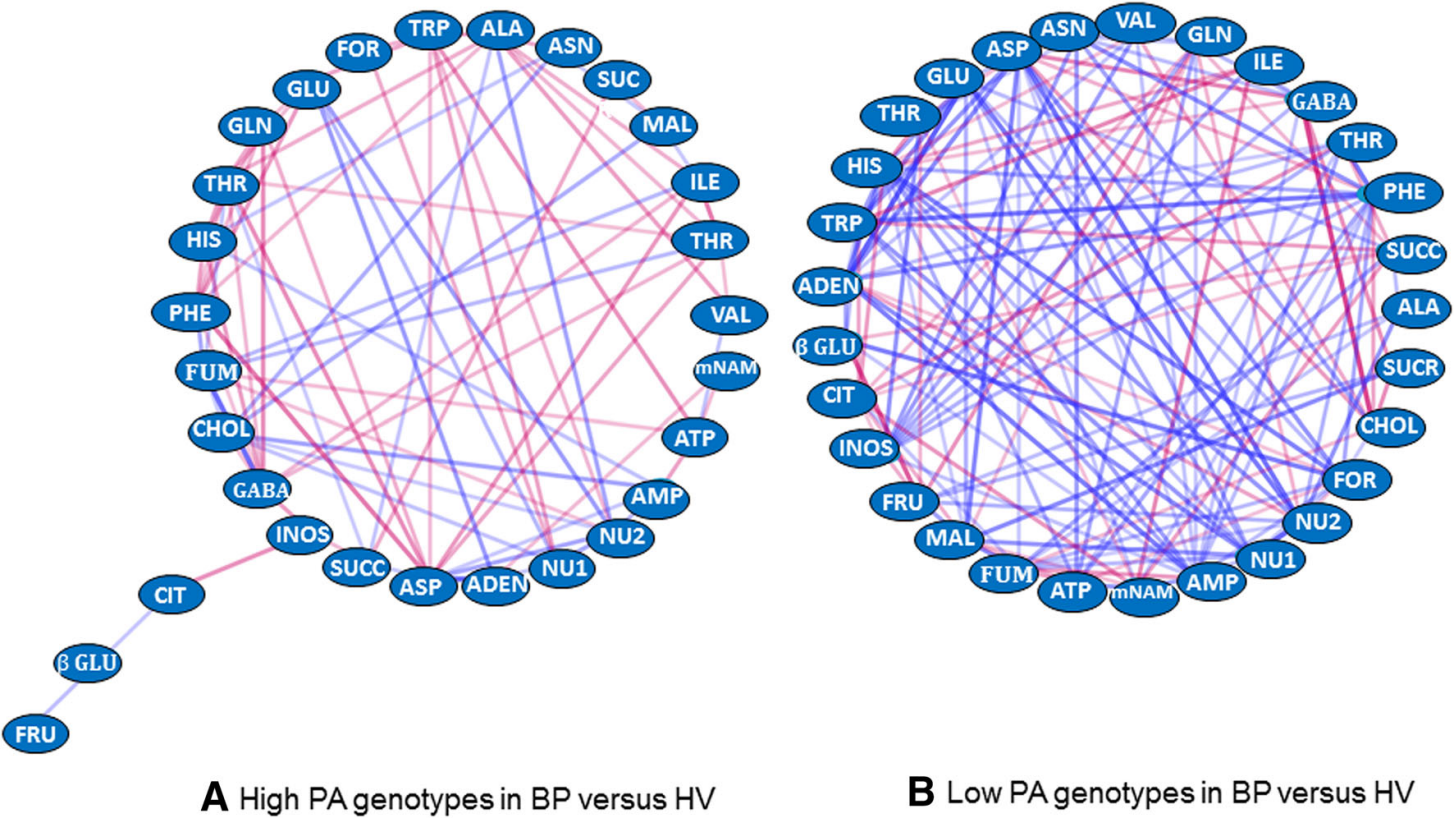
Significant changes in correlation for fruits grown with black plastic mulch versus hairy vetch mulch for high polyamine content genotypes (**a**) or low polyamine content genotypes (**b**). Significant increases from black plastic to hairy vetch in correlation are depicted as *blue lines*, significant decreases as *red lines*, width of the line indicating the magnitude of change. Note that there are more changes for low than high polyamine content genotypes, suggesting that effects of mulch are more pronounced for low polyamine content genotypes; see text for further explanation

The univariate analysis described above showed that the HV mulching system induced a similar metabolic profile as the high polyamine genotypes 8 and 10 (Fig. 3), and we looked to the metabolic network correlations for additional evidence. For this, we examined correlations of metabolites between black polyethylene (BP) versus HV mulch; these two mulching systems had the most contrasting metabolic responses in previous analyses (Fig. 3). The significant changes in network correlations between BP and HV were identified for each of the two polyamine genotypes, high or low (Fig. 5c). Since the figures represent changes in correlation, we expected fewer changes between BP and HV mulch systems in high polyamine genotypes than for the low polyamine genotypes because the former genotypes would already have achieved the distinct metabolite profiles closer to those generated by HV mulch. Our network data supports this hypothesis showing that, within the high polyamine genotypes, the network density of metabolic changes between BP and HV (Fig. 6a) was substantially reduced compared to the more dense network of metabolic changes between BP and HV within the low polyamine genotypes (Fig. 6b).

## Discussion

We demonstrate distinct effects on fruit metabolome as affected by the abundance and deficiency of plant growth regulators such as ethylene, methyl jasmonate and polyamines (spermidine and spermine) generated via transgenic intervention, and by the agroecosystem environment. Our data on metabolic changes reinforce the need to account for agroecosystem effects on ‘novel’ and other engineered genotypes for variables like fruit yield, metabolite/nutrient content and longevity. Studies like ours contribute information necessary for agricultural sustainability by identifying genotypes that have robust agronomic and other desirable traits under different agroecosystems (Groβkinsky et al. 2015). Further, the analyses presented here on metabolic networks across genotypes and agroecosystems highlight differences in the structure of primary metabolic networks, and reveals the fluidity of plant metabolic networks. These findings concur with the recent discussion on differential metabolic networks of plant metabolism (Omranian et al. 2015).

Little information is available on the flexibility of metabolite profiles in specific genotypes when grown under environment-controlled greenhouse (GH) versus under different agroecosystems in the field. Such a comparison proved quite revealing, when data generated in the present study were compared to those using GH-grown tomatoes (Mattoo et al. 2006; Kausch et al. 2012; Sobolev et al. 2014). First, under field conditions high polyamine lines (lines 8 and 10) were richer in amino acids (GLN, ASP, THR, ASN, GLU, GABA and HIS) and energy metabolites than was found previously under GH conditions (Mattoo et al. 2006); however, organic acid levels (citrate and fumarate) in the field and greenhouse were not different. Second, fruit with the ethylene deficiency trait more severely limited the accumulation of Krebs cycle intermediates under field conditions than in the GH (Sobolev et al. 2014). Methyl jasmonate-deficient fruits were remarkably different from high polyamine fruits when grown in the greenhouse, since levels of many amino acids were decreased (Mattoo et al. 2006; Kausch et al. 2012), but, conversely, methyl jasmonate-deficient fruit accumulated higher levels of several amino acids (ALA, VAL, ASP and GLU) under field conditions. Interestingly, under field conditions, although the energy metabolites (ATP + ADP) were higher in both ethylene- and methyl jasmonate-deficient fruits, fruit from both these lines accumulated lower amounts of Krebs cycle intermediates, compared to GH conditions.

Comparative analysis of the double transgenic fruit provided additional insights on the cross-talk between high polyamine trait and that of ethylene deficiency or methyl jasmonate deficiency. The ethylene deficient trait was dominant over the high polyamine trait in decreasing the levels of amino acids, except ASN, under field conditions. In contrast, the high polyamine (Spd/Spm) trait was dominant in the background of methyl jasmonate deficiency in maintaining higher levels of several amino acids (GLN, ALA, TYR and GABA) under field conditions. Similarly, the high polyamine trait was found to be dominant for Krebs cycle intermediates—citrate, succinate and fumarate—and energy-related metabolites (ATP + ADP) under field conditions but not under greenhouse conditions. Collectively, these results affirm that specific interactions which exist between a particular metabolite pathway and growth environment are affected by the genotype being tested, and will therefore influence the metabolite quality of a crop.

Two other facts to note are: (1) the precursors of polyamine biosynthesis are themselves amino acids—arginine (ARG) and ornithine (ORN)—whose decarboxylation reactions form putrescine (Put), which is subsequently converted step-wise to spermidine (Spd), spermine (Spm) and thermo-spermine (T-Spm); (2) studies with *Arabidopsis* mutants and transgenic crops have shown involvement of polyamines in plant resistance to drought, salinity and heat (Alcázar et al. 2010; Mattoo et al. 2015). With this in mind, it is interesting that Put is also a source for another signalling and stress-related molecule GABA, which is central to the GABA shunt (Flores and Filner 1985; Shelp et al. 2012; Yang et al. 2013; Michaeli and Fromm 2015). GABA can be synthesized from GLU and its catabolism can generate GLU and ASP (Takayama and Ezura 2015). GABA also metabolizes to succinate contributing to the Krebs cycle, thus becoming an energy source. In addition, GABA can be transaminated with pyruvate to yield alanine (or with 2-oxoglutarate to yield glutamate), generating succinic semialdehyde, which can then be metabolized to succinate (Vandewalle and Olsson 1983; Patterson and Graham 1987; Shelp et al. 1999; Michaeli and Fromm 2015; Takayama and Ezura 2015). The conversion of glutamate to succinate via the action of glutamate decarboxylase, GABA transaminase, and succinic semialdehyde dehydrogenase forms the GABA shunt (see reviews, Michaeli and Fromm 2015; Takayama and Ezura 2015), affording an alternative pathway for glutamate entry into the Krebs cycle. Whether the polyamine-induced GABA shunt is a means for polyamines to promote resistance against abiotic stress is an intriguing question and needs to be further explored.

Many of these metabolites were affected by genotype in our analyses. Univariate analyses showed alanine and succinate levels were increased in high polyamine genotypes, suggesting an accelerated movement of carbon through polyamines into Krebs cycle along with generation of ATP + ADP, which were also increased in high polyamine genotypes. Network analysis also showed alanine and succinate as hubs of metabolic changes as genotype changes from low to high polyamine accumulation (Fig. 5a). Although inferring changes in metabolic rate kinetics for specific metabolic reactions is not currently possible from network correlation data (Steuer et al. 2003; Kügler and Yang 2014), it is reasonable to assume a network of positive and negative changes in metabolic kinetics as seen in Fig. 5a in response to altered activity of SAMDC in these genotypes.

Finally, our data analysis highlights the distinctive fluidity of metabolite networks associated with agroecosystem environment. Not only were metabolic profiles different in the field (this experiment) versus greenhouse (previous experiments) environments, but modification of the field agroecosystem had a profound effect on metabolites. The traditional culture of tomatoes in black polyethylene mulch increases soil temperature, and accelerates vegetative growth and the advent of reproductive development leading to higher relative growth rates of fruit (Teasdale and Abdul-Baki 1997). In contrast, tomatoes grown in hairy vetch cover crop residue are exposed to a soil environment with cooler temperatures, higher nitrogen mineralization, and an altered microbial community (Buyer et al. 2010), leading to a higher ratio of vegetative to fruit growth rate and a longer growth and fruiting period than tomatoes grown with black polyethylene (Teasdale and Abdul-Baki 1997). Metabolic profiles depicted in univariate analyses in this paper show differential metabolic patterns in fruit grown in these two distinct agroecosystem mulching environments. Higher levels of selected amino and organic acids and lower sugar levels reflect the more vigorous vegetative growth of HV-grown compared to BP-grown tomatoes, as well as changes in the covariances among metabolites, shown in the network analysis. Previous research has shown positive correlations between amino acids and carboxylic acids, and negative correlations with sugars, in tomato seed and fruit tissues (Toubiana et al. 2012). These authors suggest that these associations are evidence of cross-talk between C and N networks in response to changes in resource competition between plant vegetative and reproductive growth. It is intriguing that these metabolic shifts in response to mulching environment are similar to shifts in response to high polyamine genotypes. This similarity is highlighted by the distinct loss in density of metabolic correlation changes between HV and BP when compared within high-polyamine genotypes than within low-polyamine genotypes (Fig. 6).

## Conclusions

This study portrays how metabolite relationships change under different genotypic and environmental conditions. Although these networks are surprisingly dynamic, examples of selectively conserved associations were evident. The importance of the specific agroecosystem environment in metabolomics research is highlighted; metabolic profiles are highly sensitive to the nature of the growing conditions under which tomato plants are grown and fruit is ripened. The development of transgenic germplasm will be subject to the same genotype by environment interactions that traditional breeders have addressed for centuries. Future research is needed to identify other specific traits that modulate metabolomic profiles in fruit and better define how expressions of these traits are modified by agroecosystem environment.

## Electronic supplementary material

Below is the link to the electronic supplementary material.

Supplementary material 1 (DOC 1623 kb)

## Abbreviations

ADEN: Adenosine
ANOVA: Analysis of variance
BP: Black polyethylene
BS: Bare soil
C: Carbon
CCA: Canonical correlation analysis
CO_2_: Carbon dioxide
G × E: Genetic background × ecosystem environment
GH: Greenhouse
HV: Hairy vetch
*meJAS*: Methyl jasmonate
mNAM: Methyl nicotinamide
N: Nitrogen
NMR: Nuclear magnetic resonance
PCA: Principal components analysis
Put: Putrescine
Q-PCR: Quantitative polymerase chain reaction
RY: Rye
*SlE8*: Tomato E8 gene
*SlLOX*: Tomato lipoxygenase gene
Spd: Spermidine
Spm: Spermine
WCNA: Weighted correlation network analysis
ySAMDC: Yeast SAM decarboxylase
