# Supplementary material for: Fruit metabolite networks in engineered and non-engineered tomato genotypes reveal fluidity in a hormone and agroecosystem specific manner
Source: Metabolomics. 2016 May 11;12:103. doi: 10.1007/s11306-016-1037-2 (PMC4869742; doi:10.1007/s11306-016-1037-2)

**Electronic supplementary material – On Line Resource**

**Fruit metabolite networks in engineered and non-engineered tomato genotypes reveal fluidity in a hormone and agroecosystem specific manner**

Tahira Fatima^1,6^, Anatoly P. Sobolev^2^, John R. Teasdale^1^, Matthew Kramer^3^, Jim Bunce^4^, Avtar K. Handa^5^, and Autar K. Mattoo^1*^

^1^ Sustainable Agricultural Systems, ^3^ Statistics Group, and ^4^ Crop Systems Laboratories, United States Department of Agriculture, Agricultural Research Service, The Henry A. Wallace Beltsville Agricultural Research Center, Beltsville, MD 20705, USA; ^2^ Magnetic Resonance Laboratory “Annalaura Segre”, Institute of Chemical Methodologies, CNR, Monterotondo (Rome), Italy; ^5^ Department of Horticulture and Landscape Architecture, Purdue University, West Lafayette, Indiana 47907-2010, USA

^6^ **Present address**: University of Western Ontario, London, Canada

***Corresponding author**: Email: [autar.mattoo@ars.usda.gov](mailto:autar.mattoo@ars.usda.gov )

Tel: 301 504 6622; Fax: +1 301 504 6491

**Supplementary Table 1**. Codes and descriptions of tomato genotypes

| Code | Genotype | Description |
| --- | --- | --- |
| 5 | 556AZ | Azygous control line (ref. Mehta et al. 2002) |
| 8 | 556HO | Accumulates polyamines spermidine and spermine (ref. Mehta et al. 2002) |
| 10 | 579HO | Accumulates polyamines spermidine and spermine (ref. Mehta et al. 2002) |
| 12 | 650-12HO | Suppressed lipoxygenase, deficient in methyl jasmonate (ref. Kausch et al. 2012) |
| 20 | LS-4HO | Backcross between 556HO and 650-12HO  (present investigation) |
| 2 | 2AS-2HO | Suppressed ACS2 gene expression, reduced ethylene by 50% (ref. Sobolev et al. 2014) |
| 4 | 102AS-1HO | Backcross between 579HO and 2AS-2HO (ref. Sobolev et al. 2014) |

**Supplementary Table 2**. Names and abbreviations of metabolites

| Metabolite class | Metabolite | Abbreviation |
| --- | --- | --- |
| Polyamines | Putrescine | Put |
|  | Spermidine | Spd |
|  | Spermine | Spm |
| Amino acids | Alanine | Ala |
|  | Asparagine | Asn |
|  | Aspartate | Asp |
|  | GABA | GABA |
|  | Glutamine | Glu |
|  | Glycine | Gly |
|  | Histidine | His |
|  | Isoleucine | Ile |
|  | Phenylalanine | Phe |
|  | Threonine | Thr |
|  | Tryptophan | Trp |
|  | Tyrosine | Tyr |
|  | Valine | Val |
| Organic acids | Citrate | Citr |
|  | Formate | Form |
|  | Fumarate | Fum |
|  | Malate | Mal |
|  | Succinate | Succ |
| Sugars | β-Glucose | bGlc |
|  | Fructose | Fruc |
|  | Inositol | Inos |
|  | Sucrose | Sucr |
| Energy | Adenosine | Aden |
|  | AMP | AMP |
|  | ATP+ADP | ATP+ADP |
|  | B | B |
|  | Choline | Chol |
|  |  |  |
| Other | Nucl1 | Nucl1 |
|  | Nucl2 | Nucl2 |
|  | methyl nicotinamide | mNAM |

**Supplementary Table 3**. Codes, descriptions, sources, accessions of genes and primers.

| **qPCR symbol** | **Gene (symbol)** | **Source** | **Accession #** | **Primer combination** |
| --- | --- | --- | --- | --- |
| ACT | Actin-7 (ACT) | Tomato | AB199316 | **Forward (5’-3’)**  ATGCGTATGTGGGTGATGAA **Reverse (5’-3’)** GCCTCAGTCAGGAGAACAGG |
| E8 | Ethylene-responsive fruit ripening gene E8 promoter (E8) | Tomato | AF515784 | **Forward (5’-3’)**  TGTCTCTTTCTTGTTCCCATTTC **Reverse (5’-3’)** AAATCTCAATATGAGGATGCCA |
| SAMDC | S-adenosyl-methionine decarboxylase (SPE2) | Yeast | M38434 | **Forward (5’-3’)**  CTGGATGCCTTTTTGTTGAG **Reverse (5’-3’)** TACTTGCCCCCTTGTGTTGT |
| LOXB | Lipoxygenase (LOX) | Tomato | U13681 | **Forward (5’-3’)**  GGCGACAAGAAAGATGAGG **Reverse (5’-3’)** AAAGTAGGGCGATTAGGGAGA |
| ACS2 | 1-aminocyclopropane-1-carboxylate synthase (LE-ACC2) | Tomato | X59145 | **Forward (5’-3’)**  TTCGGAGGTTCGTAGGTGTT **Reverse (5’-3’)** TGGTGAGGGAGGAATAGGTG |

**Supplementary Table 4.** Polyamine content (nmol g^-1^ DW) of pink and red tomato fruit.

| Genotype code | Putrescine | Spermidine | Spermine |
| --- | --- | --- | --- |
| 5 (wild type) | 864 a | 388 b | 15 c |
|  |  |  |  |
| 8 (high polyamine) | 289 b | 923 a | 60 b |
| 12 (low me-jas) | 1005 a | 252 b | 10 c |
| 20 (8 x 12 cross) | 177 b | 853 a | 114 a |
| 10 (high polyamine) | 154 b | 1062 a | 49 b |
| 2 (low ethylene) | 730 a | 298 b | 13 c |
| 4 (10 x 2 cross) | 132 b | 901 a | 54 b |

Values are least square means across two years, four mulching treatments, and two ripening stages. Values in columns followed by the same letter are not significantly different (*P* <0.05).

**Supplementary Table 5.** Photosynthesis, stomatal conductance, internal leaf CO_2_ concentration, and fruit yield of wild type (line 5), high polyamine (line 10), low ethylene (line 2), and high polyamine x low ethylene (line 4) tomato genotypes.

| Genotype | Net photosynthesis  (µmol m^-2^ s^-1^) | Stomatal conductance  (mmol m^-2^ s^-1^) | Internal CO_2_  (µmol mol^-1^) | Fruit yield  (kg plant^-1^) |
| --- | --- | --- | --- | --- |
| 5 | 29.9 bc | 982 bc | 292 a | 6.95 a |
| 10 | 28.7 c | 933 c | 293 a | 4.60 b |
| 4 | 30.5 ab | 1025 ab | 292 a | 7.13 a |
| 2 | 31.7 a | 1099 a | 294 a | 7.54 a |

Values in columns followed by the same letter are not significantly different (*P* <0.05).

**Supplementary Table 6**. Univariate analyses of tomato fruit metabolite response to genotype. Wild type, high polyamine, methyl jasmonate deficient, ethylene deficient genotypes, and selected crosses are grouped with their crossing partners. Data are the least square means of four mulch treatments, two fruit ripening stages (pink and red), and two years. Values followed by the same letter within columns are not significantly different (P<0.05). Values with no letters indicate a non-significant analysis of variance.

A. Amino Acids

| Genotype | Ala | Asn | Asp | GABA | Glu | Gly | His |
| --- | --- | --- | --- | --- | --- | --- | --- |
| 5 (wild type) | 39.9 ab | 39.7 c | 51.9 | 145 | 57.5 | 87.8 | 6.98 |
| 8 (high polyamine) | 50.7 a | 48.1 a | 59.1 | 153 | 66.7 | 94.8 | 7.72 |
| 12 (me-jas deficient) | 43.1 ab | 37.0 c | 64.5 | 150 | 67.8 | 76.2 | 7.48 |
| 20 (8 x 12) | 52.8 a | 41.2 bc | 51.5 | 158 | 53.1 | 84.9 | 6.93 |
| 10 (high polyamine) | 31.8 bc | 46.6 ab | 53.6 | 173 | 66.6 | 97.1 | 7.94 |
| 2 (ethylene deficient) | 7.5 d | 37.8 c | 53.9 | 147 | 66.0 | 83.9 | 7.23 |
| 4 (10 x 2) | 23.7 cd | 43.4 abc | 46.1 | 136 | 53.8 | 86.4 | 6.44 |
|  | Iso | Phe | Thr | Try | Tyr | Val | Mean |
| 5 (wild type) | 13.1 | 16.7 | 51.2 | 1.59 | 6.12 | 10.4 ab | -0.050 b |
| 8 (high polyamine) | 14.9 | 18.6 | 56.1 | 1.61 | 7.58 | 10.1 ab | +0.293 a |
| 12 (me jas deficient) | 12.7 | 16.7 | 53.2 | 1.58 | 5.94 | 11.5 a | +0.003 b |
| 20 (8 x 12) | 13.6 | 16.7 | 49.0 | 1.62 | 6.83 | 9.1 bc | -0.009 b |
| 10 (high polyamine) | 13.6 | 16.6 | 53.5 | 1.55 | 6.63 | 7.5 cd | +0.095 ab |
| 2 (ethylene deficient) | 12.5 | 18.0 | 48.2 | 1.71 | 6.75 | 10.6 ab | -0.060 b |
| 4 (10 x 2) | 10.6 | 13.2 | 38.8 | 1.50 | 4.99 | 6.2 d | -0.464 c |

B. Organic Acids

| Genotype | Citrate | Formate | Fumarate | Malate | Succinate | Mean |
| --- | --- | --- | --- | --- | --- | --- |
| 5 (wild type) | 658 bc | 2.00 | 6.99 | 38.4 | 42.9 bc | +0.027 ab |
| 8 (high polyamine) | 718 ab | 2.11 | 7.45 | 43.7 | 47.2 b | +0.302 a |
| 12 (me jas deficient) | 562 d | 2.58 | 4.51 | 30.2 | 38.5 c | -0.329 b |
| 20 (8 x 12) | 687 b | 1.67 | 6.51 | 36.6 | 48.3 b | +0.001 ab |
| 10 (high polyamine) | 779 a | 2.08 | 7.17 | 37.0 | 56.5 a | +0.371 a |
| 2 (ethylene deficient) | 574 cd | 1.99 | 5.85 | 32.4 | 41.8 bc | -0.276 b |
| 4 (10 x 2) | 682 b | 1.72 | 6.99 | 34.7 | 50.9 ab |  |

C. Sugars

| Genotype | βGlucose | Fructose | Inositol | Sucrose | Mean |
| --- | --- | --- | --- | --- | --- |
| 5 (wild type) | 554 | 578 | 10.5 ab | 4.37 | +0.226 |
| 8 (high polyamine) | 516 | 557 | 9.4 bc | 3.51 | -0.103 |
| 12 (me jas deficient) | 558 | 566 | 9.9 abc | 1.20 | +0.025 |
| 20 (8 x 12) | 552 | 595 | 8.3 c | 1.45 | -0.022 |
| 10 (high polyamine) | 555 | 560 | 9.3 bc | 6.12 | +0.151 |
| 2 (ethylene deficient) | 551 | 582 | 11.7 a | 1.93 | +0.223 |
| 4 (10 x 2) | 547 | 612 | 9.2 bc | 1.85 | +0.102 |

D. Energy Metabolites

| Genotype | Adenosine | AMP | ATP+ADP | B | Choline | Mean |
| --- | --- | --- | --- | --- | --- | --- |
| 5 (wild type) | 3.85 b | 4.58 | 1.78 bc | 22.7 | 154 b | -0.109 |
| 8 (high polyamine) | 2.80 cd | 5.62 | 2.17 ab | 25.0 | 165 b | +0.148 |
| 12 (me jas deficient) | 3.92 b | 4.71 | 1.49 c | 22.2 | 152 b | -0.202 |
| 20 (8 x 12) | 2.71 d | 5.10 | 1.92 abc | 19.2 | 162 b | -0.172 |
| 10 (high polyamine) | 2.73 cd | 5.31 | 2.08 ab | 22.0 | 192 a | +0.196 |
| 2 (ethylene deficient) | 6.30 a | 5.47 | 1.99 abc | 28.0 | 148 b | +0.448 |
| 4 (10 x 2) | 3.60 bc | 4.93 | 2.24 a | 18.4 | 153 b | -0.075 |

E. Other Metabolites

| Genotype | Nucl1 | Nucl2 | mNAM |
| --- | --- | --- | --- |
| 5 (wild type) | 0.899 | 3.68 | 1.81 b |
| 8 (high polyamine) | 0.966 | 3.61 | 1.88 b |
| 12 (me jas deficient) | 1.044 | 3.81 | 1.79 b |
| 20 (8 x 12) | 0.882 | 3.66 | 1.87 b |
| 10 (high polyamine) | 0.846 | 3.32 | 2.49 a |
| 2 (ethylene deficient) | 0.881 | 4.29 | 1.92 b |
| 4 (10 x 2) | 0.853 | 3.99 | 1.99 b |

**Supplementary Table 7.** Univariate analyses of tomato fruit metabolite response to mulch. Data are the least square means of seven genotype treatments, two fruit ripening stages (pink and red), and two years. Values followed by the same letter within columns are not significantly different (P<0.05). Values with no letters indicate a non-significant analysis of variance.

1. Amino Acids.

| Mulch | Ala | Asn | Asp | GABA | Glu | Gly | His |
| --- | --- | --- | --- | --- | --- | --- | --- |
| Black polyethylene | 39.6 | 39.6 | 46.8 b | 151 b | 50.9 b | 81.4 | 6.95 |
| Bare soil | 31.0 | 44.6 | 56.8 ab | 129 b | 61.9 ab | 86.4 | 7.70 |
| Hairy vetch | 32.1 | 42.5 | 55.1 ab | 174 a | 67.9 a | 93.2 | 7.27 |
| Rye | 39.9 | 41.2 | 58.8 a | 153 ab | 65.8 a | 88.2 | 7.06 |
|  |  |  |  |  |  |  |  |
|  | Iso | Phe | Thr | Try | Tyr | Val | Mean |
| Black polyethylene | 12.5 | 14.8 | 45.6 b | 1.51 | 5.90 | 9.3 | -0.227 c |
| Bare soil | 12.2 | 17.1 | 43.0 b | 1.71 | 6.71 | 9.0 | -0.049 b |
| Hairy vetch | 13.9 | 17.6 | 56.4 a | 1.64 | 6.53 | 10.1 | +0.150 a |
| Rye | 13.4 | 17.2 | 55.0 a | 1.52 | 6.49 | 9.0 | +0.016 ab |

1. Organic Acids.

| Mulch | Citrate | Formate | Fumarate | Malate | Succinate | Mean |
| --- | --- | --- | --- | --- | --- | --- |
| Black polyethylene | 617 c | 1.92 | 6.39 | 31.3 b | 45.5 b | -0.168 b |
| Bare soil | 606 c | 2.18 | 5.50 | 34.4 ab | 44.5 b | -0.146 b |
| Hairy vetch | 765 a | 2.05 | 7.72 | 41.4 a | 52.6 a | +0.402 a |
| Rye | 676 b | 1.93 | 6.37 | 37.4 ab | 43.8 b | -0.012 b |

1. Sugars.

| Mulch | βGlucose | Fructose | Inositol | Sucrose | Mean |
| --- | --- | --- | --- | --- | --- |
| Black polyethylene | 586 a | 595 a | 10.0 | 2.29 | +0.278 |
| Bare soil | 569 a | 634 a | 9.5 | 3.74 | +0.361 |
| Hairy vetch | 514 b | 553 b | 9.7 | 0.73 | -0.218 |
| Rye | 520 b | 532 b | 9.8 | 4.91 | -0.077 |

1. Energy Metabolites.

| Mulch | Adenosine | AMP | ATP+ADP | B | Choline | Mean |
| --- | --- | --- | --- | --- | --- | --- |
| Black polyethylene | 3.81 | 4.63 | 2.01 | 20.4 | 156 | -0.080 |
| Bare soil | 4.01 | 5.02 | 2.01 | 25.4 | 163 | +0.173 |
| Hairy vetch | 3.68 | 5.42 | 1.97 | 23.1 | 165 | +0.120 |
| Rye | 3.30 | 5.34 | 1.82 | 21.2 | 159 | -0.080 |

1. Other Metabolites.

| Mulch | Nucl1 | Nucl2 | mNAM |
| --- | --- | --- | --- |
| Black polyethylene | 0.812 | 3.84 ab | 1.81 b |
| Bare soil | 0.998 | 4.22 a | 1.91 b |
| Hairy vetch | 0.974 | 3.54 b | 2.29 a |
| Rye | 0.856 | 3.46 b | 1.85 b |

**Supplementary Table 8.** Correlation statistics for each pair of metabolites. The correlations were calculated as follows. First, the data were sliced into various (overlapping) partitions: type = ON, OFF; stage = R, P; genotype = 2, 4, 5, 8, 10, 12, 20; mulch = B, BP, HV, RY; year = 2006, 2007. For each slice, a Pearson correlation was calculated for the metabolite pair. This was transformed to a *Z* score using Fisher's *Z* transformation. The mean and standard deviation of these transformed correlations was calculated (columns 3 and 4), and the mean back-transformed to the proportion scale for column 2.

**Metabolite Pair MeanCorr Z SD_Z**

ILE-VAL 0.914 1.552 0.396

ILE-THR 0.913 1.545 0.273

ILE-ALA -0.051 -0.051 0.268

ILE-GLU 0.231 0.235 0.208

ILE-GLN 0.854 1.272 0.284

ILE-ASP 0.286 0.294 0.241

ILE-ASN 0.749 0.971 0.273

ILE-TYR 0.877 1.363 0.410

ILE-HIS 0.730 0.929 0.238

ILE-PHE 0.617 0.720 0.254

ILE-TRP 0.685 0.839 0.231

ILE-GABA 0.622 0.729 0.256

ILE-CITRIC 0.306 0.316 0.176

ILE-SUCC 0.553 0.623 0.375

ILE-MALIC 0.261 0.267 0.144

ILE-FUMARIC 0.184 0.186 0.215

ILE-Formic -0.070 -0.070 0.156

ILE-bGLC -0.313 -0.324 0.224

ILE-FRUCT -0.263 -0.269 0.209

ILE-SUCR 0.092 0.093 0.172

ILE-INOS 0.552 0.621 0.322

ILE-B 0.818 1.151 0.372

ILE-CHOLINE 0.571 0.649 0.205

ILE-Aden -0.113 -0.113 0.193

ILE-AMP -0.152 -0.153 0.296

ILE-ATP+ADP -0.131 -0.131 0.230

ILE-Nucl1 0.091 0.091 0.162

ILE-Nucl2 -0.198 -0.201 0.269

ILE-Trigon 0.596 0.686 0.318

VAL-THR 0.803 1.108 0.265

VAL-ALA 0.003 0.003 0.239

VAL-GLU 0.191 0.193 0.195

VAL-GLN 0.716 0.899 0.355

VAL-ASP 0.252 0.258 0.220

VAL-ASN 0.645 0.766 0.330

VAL-TYR 0.744 0.959 0.358

VAL-HIS 0.664 0.799 0.289

VAL-PHE 0.482 0.525 0.209

VAL-TRP 0.656 0.786 0.238

VAL-GABA 0.484 0.528 0.264

VAL-CITRIC 0.102 0.102 0.125

VAL-SUCC 0.358 0.375 0.400

VAL-MALIC 0.135 0.136 0.143

VAL-FUMARIC 0.077 0.077 0.202

VAL-Formic -0.067 -0.067 0.163

VAL-bGLC -0.269 -0.276 0.228

VAL-FRUCT -0.218 -0.222 0.227

VAL-SUCR 0.059 0.059 0.157

VAL-INOS 0.509 0.562 0.361

VAL-B 0.771 1.022 0.361

VAL-CHOLINE 0.435 0.467 0.222

VAL-Aden 0.016 0.016 0.171

VAL-AMP -0.217 -0.221 0.254

VAL-ATP+ADP -0.237 -0.242 0.177

VAL-Nucl1 0.102 0.102 0.192

VAL-Nucl2 -0.125 -0.126 0.232

VAL-Trigon 0.512 0.565 0.372

THR-ALA 0.078 0.078 0.244

THR-GLU 0.495 0.543 0.236

THR-GLN 0.887 1.406 0.223

THR-ASP 0.526 0.585 0.232

THR-ASN 0.798 1.094 0.212

THR-TYR 0.870 1.335 0.310

THR-HIS 0.841 1.224 0.207

THR-PHE 0.727 0.923 0.206

THR-TRP 0.695 0.857 0.259

THR-GABA 0.646 0.768 0.179

THR-CITRIC 0.339 0.353 0.150

THR-SUCC 0.428 0.457 0.323

THR-MALIC 0.188 0.191 0.149

THR-FUMARIC 0.106 0.106 0.224

THR-Formic 0.084 0.084 0.157

THR-bGLC -0.391 -0.413 0.207

THR-FRUCT -0.365 -0.383 0.194

THR-SUCR -0.017 -0.017 0.109

THR-INOS 0.512 0.566 0.299

THR-B 0.785 1.058 0.320

THR-CHOLINE 0.592 0.681 0.225

THR-Aden 0.034 0.034 0.187

THR-AMP 0.036 0.036 0.275

THR-ATP+ADP -0.004 -0.004 0.215

THR-Nucl1 0.327 0.340 0.187

THR-Nucl2 -0.025 -0.025 0.275

THR-Trigon 0.627 0.737 0.274

ALA-GLU 0.307 0.317 0.312

ALA-GLN -0.096 -0.096 0.165

ALA-ASP 0.370 0.389 0.321

ALA-ASN -0.036 -0.036 0.249

ALA-TYR 0.128 0.129 0.215

ALA-HIS 0.131 0.132 0.245

ALA-PHE 0.350 0.365 0.245

ALA-TRP -0.085 -0.085 0.171

ALA-GABA 0.115 0.115 0.233

ALA-CITRIC 0.012 0.012 0.175

ALA-SUCC -0.469 -0.509 0.176

ALA-MALIC 0.238 0.243 0.423

ALA-FUMARIC 0.148 0.149 0.366

ALA-Formic 0.087 0.087 0.218

ALA-bGLC 0.266 0.273 0.307

ALA-FRUCT 0.211 0.214 0.293

ALA-SUCR -0.195 -0.197 0.179

ALA-INOS -0.357 -0.374 0.192

ALA-B -0.352 -0.368 0.113

ALA-CHOLINE 0.212 0.215 0.228

ALA-Aden 0.223 0.227 0.443

ALA-AMP 0.569 0.646 0.279

ALA-ATP+ADP 0.389 0.411 0.218

ALA-Nucl1 0.334 0.347 0.278

ALA-Nucl2 0.671 0.813 0.364

ALA-Trigon -0.190 -0.192 0.125

GLU-GLN 0.438 0.470 0.209

GLU-ASP 0.913 1.545 0.165

GLU-ASN 0.578 0.659 0.255

GLU-TYR 0.249 0.254 0.171

GLU-HIS 0.754 0.983 0.265

GLU-PHE 0.421 0.449 0.217

GLU-TRP 0.467 0.506 0.242

GLU-GABA 0.276 0.284 0.216

GLU-CITRIC -0.001 -0.001 0.246

GLU-SUCC -0.290 -0.298 0.292

GLU-MALIC -0.272 -0.279 0.219

GLU-FUMARIC -0.333 -0.346 0.281

GLU-Formic 0.605 0.701 0.217

GLU-bGLC -0.534 -0.595 0.162

GLU-FRUCT -0.427 -0.456 0.149

GLU-SUCR -0.140 -0.141 0.210

GLU-INOS 0.205 0.208 0.186

GLU-B 0.302 0.311 0.218

GLU-CHOLINE 0.462 0.499 0.215

GLU-Aden 0.590 0.678 0.350

GLU-AMP 0.398 0.421 0.249

GLU-ATP+ADP 0.222 0.226 0.168

GLU-Nucl1 0.853 1.267 0.192

GLU-Nucl2 0.518 0.574 0.324

GLU-Trigon 0.495 0.543 0.243

GLN-ASP 0.421 0.449 0.181

GLN-ASN 0.899 1.465 0.299

GLN-TYR 0.777 1.037 0.332

GLN-HIS 0.815 1.143 0.209

GLN-PHE 0.608 0.705 0.233

GLN-TRP 0.627 0.736 0.233

GLN-GABA 0.575 0.655 0.214

GLN-CITRIC 0.402 0.426 0.219

GLN-SUCC 0.561 0.634 0.337

GLN-MALIC 0.154 0.155 0.174

GLN-FUMARIC 0.102 0.103 0.241

GLN-Formic 0.096 0.097 0.171

GLN-bGLC -0.383 -0.403 0.235

GLN-FRUCT -0.298 -0.308 0.205

GLN-SUCR 0.136 0.137 0.125

GLN-INOS 0.536 0.598 0.236

GLN-B 0.789 1.069 0.273

GLN-CHOLINE 0.580 0.663 0.200

GLN-Aden 0.017 0.017 0.194

GLN-AMP -0.003 -0.003 0.210

GLN-ATP+ADP 0.115 0.115 0.234

GLN-Nucl1 0.246 0.251 0.137

GLN-Nucl2 -0.105 -0.106 0.209

GLN-Trigon 0.701 0.869 0.258

ASP-ASN 0.537 0.600 0.204

ASP-TYR 0.314 0.325 0.194

ASP-HIS 0.718 0.903 0.188

ASP-PHE 0.463 0.502 0.210

ASP-TRP 0.416 0.442 0.222

ASP-GABA 0.267 0.274 0.198

ASP-CITRIC 0.011 0.011 0.205

ASP-SUCC -0.266 -0.273 0.266

ASP-MALIC -0.193 -0.195 0.219

ASP-FUMARIC -0.423 -0.451 0.254

ASP-Formic 0.566 0.641 0.159

ASP-bGLC -0.547 -0.615 0.190

ASP-FRUCT -0.491 -0.537 0.195

ASP-SUCR -0.177 -0.179 0.227

ASP-INOS 0.243 0.248 0.214

ASP-B 0.297 0.306 0.231

ASP-CHOLINE 0.450 0.485 0.222

ASP-Aden 0.550 0.619 0.285

ASP-AMP 0.301 0.310 0.241

ASP-ATP+ADP 0.071 0.071 0.193

ASP-Nucl1 0.849 1.252 0.195

ASP-Nucl2 0.521 0.578 0.344

ASP-Trigon 0.373 0.392 0.192

ASN-TYR 0.633 0.747 0.294

ASN-HIS 0.881 1.380 0.221

ASN-PHE 0.459 0.496 0.281

ASN-TRP 0.676 0.822 0.250

ASN-GABA 0.379 0.399 0.227

ASN-CITRIC 0.143 0.144 0.231

ASN-SUCC 0.346 0.361 0.385

ASN-MALIC -0.039 -0.039 0.202

ASN-FUMARIC -0.033 -0.033 0.240

ASN-Formic 0.285 0.293 0.240

ASN-bGLC -0.538 -0.602 0.230

ASN-FRUCT -0.394 -0.416 0.198

ASN-SUCR 0.040 0.040 0.147

ASN-INOS 0.408 0.433 0.228

ASN-B 0.787 1.064 0.323

ASN-CHOLINE 0.520 0.576 0.215

ASN-Aden 0.188 0.190 0.270

ASN-AMP -0.010 -0.010 0.250

ASN-ATP+ADP 0.058 0.058 0.219

ASN-Nucl1 0.426 0.455 0.190

ASN-Nucl2 0.094 0.094 0.271

ASN-Trigon 0.725 0.918 0.344

TYR-HIS 0.665 0.801 0.195

TYR-PHE 0.823 1.167 0.177

TYR-TRP 0.610 0.709 0.202

TYR-GABA 0.601 0.694 0.180

TYR-CITRIC 0.411 0.437 0.175

TYR-SUCC 0.479 0.522 0.294

TYR-MALIC 0.391 0.413 0.177

TYR-FUMARIC 0.282 0.290 0.220

TYR-Formic -0.088 -0.088 0.118

TYR-bGLC -0.133 -0.134 0.206

TYR-FRUCT -0.088 -0.089 0.251

TYR-SUCR -0.027 -0.027 0.145

TYR-INOS 0.398 0.422 0.246

TYR-B 0.697 0.861 0.319

TYR-CHOLINE 0.602 0.696 0.250

TYR-Aden -0.096 -0.097 0.152

TYR-AMP 0.041 0.041 0.272

TYR-ATP+ADP 0.110 0.111 0.281

TYR-Nucl1 0.112 0.113 0.159

TYR-Nucl2 -0.026 -0.026 0.233

TYR-Trigon 0.472 0.512 0.218

HIS-PHE 0.615 0.718 0.199

HIS-TRP 0.772 1.025 0.198

HIS-GABA 0.546 0.613 0.238

HIS-CITRIC 0.110 0.110 0.152

HIS-SUCC 0.186 0.188 0.336

HIS-MALIC -0.083 -0.083 0.194

HIS-FUMARIC -0.132 -0.133 0.264

HIS-Formic 0.339 0.353 0.226

HIS-bGLC -0.495 -0.542 0.240

HIS-FRUCT -0.351 -0.367 0.206

HIS-SUCR -0.057 -0.057 0.145

HIS-INOS 0.455 0.490 0.304

HIS-B 0.748 0.967 0.300

HIS-CHOLINE 0.664 0.800 0.241

HIS-Aden 0.319 0.331 0.251

HIS-AMP 0.119 0.119 0.254

HIS-ATP+ADP 0.089 0.089 0.219

HIS-Nucl1 0.626 0.734 0.191

HIS-Nucl2 0.270 0.276 0.282

HIS-Trigon 0.744 0.959 0.288

PHE-TRP 0.436 0.467 0.178

PHE-GABA 0.685 0.839 0.158

PHE-CITRIC 0.459 0.496 0.243

PHE-SUCC 0.239 0.243 0.233

PHE-MALIC 0.437 0.468 0.288

PHE-FUMARIC 0.319 0.331 0.263

PHE-Formic -0.026 -0.026 0.178

PHE-bGLC 0.062 0.062 0.197

PHE-FRUCT 0.074 0.074 0.234

PHE-SUCR -0.085 -0.085 0.165

PHE-INOS 0.308 0.319 0.282

PHE-B 0.405 0.429 0.229

PHE-CHOLINE 0.583 0.668 0.236

PHE-Aden 0.094 0.095 0.223

PHE-AMP 0.455 0.491 0.277

PHE-ATP+ADP 0.356 0.372 0.282

PHE-Nucl1 0.271 0.278 0.187

PHE-Nucl2 0.210 0.213 0.305

PHE-Trigon 0.319 0.330 0.123

TRP-GABA 0.398 0.421 0.157

TRP-CITRIC 0.055 0.055 0.167

TRP-SUCC 0.262 0.269 0.215

TRP-MALIC -0.088 -0.089 0.133

TRP-FUMARIC -0.026 -0.026 0.151

TRP-Formic 0.124 0.125 0.192

TRP-bGLC -0.436 -0.467 0.253

TRP-FRUCT -0.215 -0.219 0.251

TRP-SUCR -0.055 -0.055 0.142

TRP-INOS 0.418 0.445 0.262

TRP-B 0.719 0.906 0.225

TRP-CHOLINE 0.552 0.621 0.246

TRP-Aden 0.177 0.179 0.228

TRP-AMP -0.078 -0.079 0.256

TRP-ATP+ADP -0.064 -0.064 0.236

TRP-Nucl1 0.432 0.462 0.237

TRP-Nucl2 0.135 0.135 0.231

TRP-Trigon 0.691 0.849 0.268

GABA-CITRIC 0.574 0.653 0.177

GABA-SUCC 0.426 0.455 0.175

GABA-MALIC 0.369 0.387 0.189

GABA-FUMARIC 0.368 0.386 0.224

GABA-Formic -0.091 -0.091 0.157

GABA-bGLC 0.048 0.048 0.193

GABA-FRUCT 0.044 0.044 0.189

GABA-SUCR 0.048 0.048 0.155

GABA-INOS 0.425 0.454 0.306

GABA-B 0.347 0.361 0.195

GABA-CHOLINE 0.668 0.807 0.225

GABA-Aden -0.086 -0.086 0.242

GABA-AMP 0.217 0.220 0.223

GABA-ATP+ADP 0.199 0.202 0.271

GABA-Nucl1 0.154 0.155 0.154

GABA-Nucl2 -0.041 -0.041 0.271

GABA-Trigon 0.429 0.459 0.238

CITRIC-SUCC 0.472 0.512 0.174

CITRIC-MALIC 0.555 0.626 0.154

CITRIC-FUMARIC 0.401 0.424 0.182

CITRIC-Formic -0.209 -0.212 0.176

CITRIC-bGLC 0.244 0.249 0.237

CITRIC-FRUCT 0.169 0.171 0.236

CITRIC-SUCR 0.089 0.089 0.262

CITRIC-INOS 0.179 0.181 0.227

CITRIC-B 0.079 0.079 0.198

CITRIC-CHOLINE 0.469 0.508 0.172

CITRIC-Aden -0.310 -0.320 0.188

CITRIC-AMP 0.263 0.269 0.207

CITRIC-ATP+ADP 0.381 0.401 0.212

CITRIC-Nucl1 -0.058 -0.058 0.226

CITRIC-Nucl2 -0.230 -0.234 0.177

CITRIC-Trigon 0.191 0.193 0.168

SUCC-MALIC 0.330 0.343 0.232

SUCC-FUMARIC 0.419 0.446 0.252

SUCC-Formic -0.300 -0.310 0.167

SUCC-bGLC -0.035 -0.035 0.291

SUCC-FRUCT -0.010 -0.010 0.278

SUCC-SUCR 0.195 0.198 0.317

SUCC-INOS 0.407 0.432 0.234

SUCC-B 0.470 0.510 0.244

SUCC-CHOLINE 0.349 0.364 0.257

SUCC-Aden -0.453 -0.489 0.229

SUCC-AMP -0.288 -0.296 0.135

SUCC-ATP+ADP -0.041 -0.041 0.169

SUCC-Nucl1 -0.356 -0.372 0.275

SUCC-Nucl2 -0.511 -0.565 0.156

SUCC-Trigon 0.387 0.409 0.272

MALIC-FUMARIC 0.640 0.757 0.193

MALIC-Formic -0.369 -0.387 0.195

MALIC-bGLC 0.355 0.371 0.252

MALIC-FRUCT 0.296 0.305 0.238

MALIC-SUCR 0.066 0.066 0.257

MALIC-INOS -0.173 -0.175 0.208

MALIC-B -0.070 -0.070 0.214

MALIC-CHOLINE 0.257 0.263 0.239

MALIC-Aden -0.326 -0.339 0.153

MALIC-AMP 0.204 0.207 0.383

MALIC-ATP+ADP 0.173 0.175 0.310

MALIC-Nucl1 -0.264 -0.270 0.251

MALIC-Nucl2 -0.114 -0.115 0.315

MALIC-Trigon -0.024 -0.024 0.181

FUMARIC-Formic -0.407 -0.432 0.176

FUMARIC-bGLC 0.449 0.484 0.223

FUMARIC-FRUCT 0.431 0.461 0.190

FUMARIC-SUCR 0.102 0.102 0.178

FUMARIC-INOS -0.063 -0.064 0.191

FUMARIC-B -0.118 -0.119 0.202

FUMARIC-CHOLINE 0.166 0.167 0.241

FUMARIC-Aden -0.380 -0.400 0.166

FUMARIC-AMP 0.288 0.296 0.243

FUMARIC-ATP+ADP 0.426 0.454 0.208

FUMARIC-Nucl1 -0.385 -0.406 0.274

FUMARIC-Nucl2 -0.080 -0.081 0.224

FUMARIC-Trigon -0.026 -0.026 0.222

Formic-bGLC -0.551 -0.619 0.221

Formic-FRUCT -0.466 -0.505 0.170

Formic-SUCR -0.076 -0.076 0.217

Formic-INOS 0.135 0.136 0.137

Formic-B 0.117 0.117 0.176

Formic-CHOLINE 0.104 0.104 0.217

Formic-Aden 0.475 0.516 0.228

Formic-AMP 0.064 0.064 0.275

Formic-ATP+ADP -0.032 -0.032 0.201

Formic-Nucl1 0.627 0.737 0.155

Formic-Nucl2 0.314 0.325 0.219

Formic-Trigon 0.275 0.283 0.280

bGLC-FRUCT 0.882 1.385 0.260

bGLC-SUCR -0.021 -0.021 0.274

bGLC-INOS -0.330 -0.343 0.250

bGLC-B -0.505 -0.557 0.240

bGLC-CHOLINE -0.143 -0.144 0.260

bGLC-Aden -0.227 -0.231 0.239

bGLC-AMP 0.332 0.345 0.246

bGLC-ATP+ADP 0.381 0.402 0.203

bGLC-Nucl1 -0.514 -0.568 0.190

bGLC-Nucl2 0.047 0.047 0.249

bGLC-Trigon -0.427 -0.456 0.279

FRUCT-SUCR -0.041 -0.041 0.235

FRUCT-INOS -0.327 -0.340 0.244

FRUCT-B -0.378 -0.398 0.232

FRUCT-CHOLINE -0.048 -0.048 0.219

FRUCT-Aden -0.115 -0.116 0.184

FRUCT-AMP 0.340 0.354 0.271

FRUCT-ATP+ADP 0.429 0.459 0.197

FRUCT-Nucl1 -0.398 -0.421 0.159

FRUCT-Nucl2 0.174 0.175 0.226

FRUCT-Trigon -0.225 -0.228 0.249

SUCR-INOS 0.221 0.225 0.232

SUCR-B 0.081 0.082 0.141

SUCR-CHOLINE -0.070 -0.070 0.109

SUCR-Aden -0.166 -0.168 0.199

SUCR-AMP -0.119 -0.120 0.212

SUCR-ATP+ADP 0.064 0.065 0.236

SUCR-Nucl1 -0.151 -0.152 0.216

SUCR-Nucl2 -0.256 -0.262 0.203

SUCR-Trigon -0.014 -0.014 0.147

INOS-B 0.532 0.594 0.222

INOS-CHOLINE 0.252 0.258 0.254

INOS-Aden 0.001 0.001 0.148

INOS-AMP -0.212 -0.215 0.231

INOS-ATP+ADP -0.104 -0.105 0.246

INOS-Nucl1 0.109 0.109 0.172

INOS-Nucl2 -0.330 -0.342 0.219

INOS-Trigon 0.359 0.375 0.316

B-CHOLINE 0.398 0.421 0.292

B-Aden 0.072 0.072 0.225

B-AMP -0.338 -0.352 0.179

B-ATP+ADP -0.249 -0.254 0.176

B-Nucl1 0.195 0.197 0.203

B-Nucl2 -0.235 -0.239 0.177

B-Trigon 0.672 0.814 0.301

CHOLINE-Aden 0.121 0.121 0.238

CHOLINE-AMP 0.189 0.191 0.270

CHOLINE-ATP+ADP 0.210 0.213 0.216

CHOLINE-Nucl1 0.356 0.373 0.205

CHOLINE-Nucl2 0.199 0.202 0.231

CHOLINE-Trigon 0.619 0.724 0.257

Aden-AMP 0.247 0.253 0.308

Aden-ATP+ADP 0.103 0.104 0.342

Aden-Nucl1 0.591 0.679 0.284

Aden-Nucl2 0.648 0.772 0.301

Aden-Trigon 0.211 0.215 0.295

AMP-ATP+ADP 0.747 0.965 0.286

AMP-Nucl1 0.344 0.358 0.219

AMP-Nucl2 0.495 0.543 0.197

AMP-Trigon -0.062 -0.062 0.260

ATP+ADP-Nucl1 0.206 0.209 0.201

ATP+ADP-Nucl2 0.445 0.478 0.179

ATP+ADP-Trigon 0.079 0.079 0.275

Nucl1-Nucl2 0.596 0.687 0.268

Nucl1-Trigon 0.391 0.413 0.206

Nucl2-Trigon 0.010 0.010 0.230

**Figures**

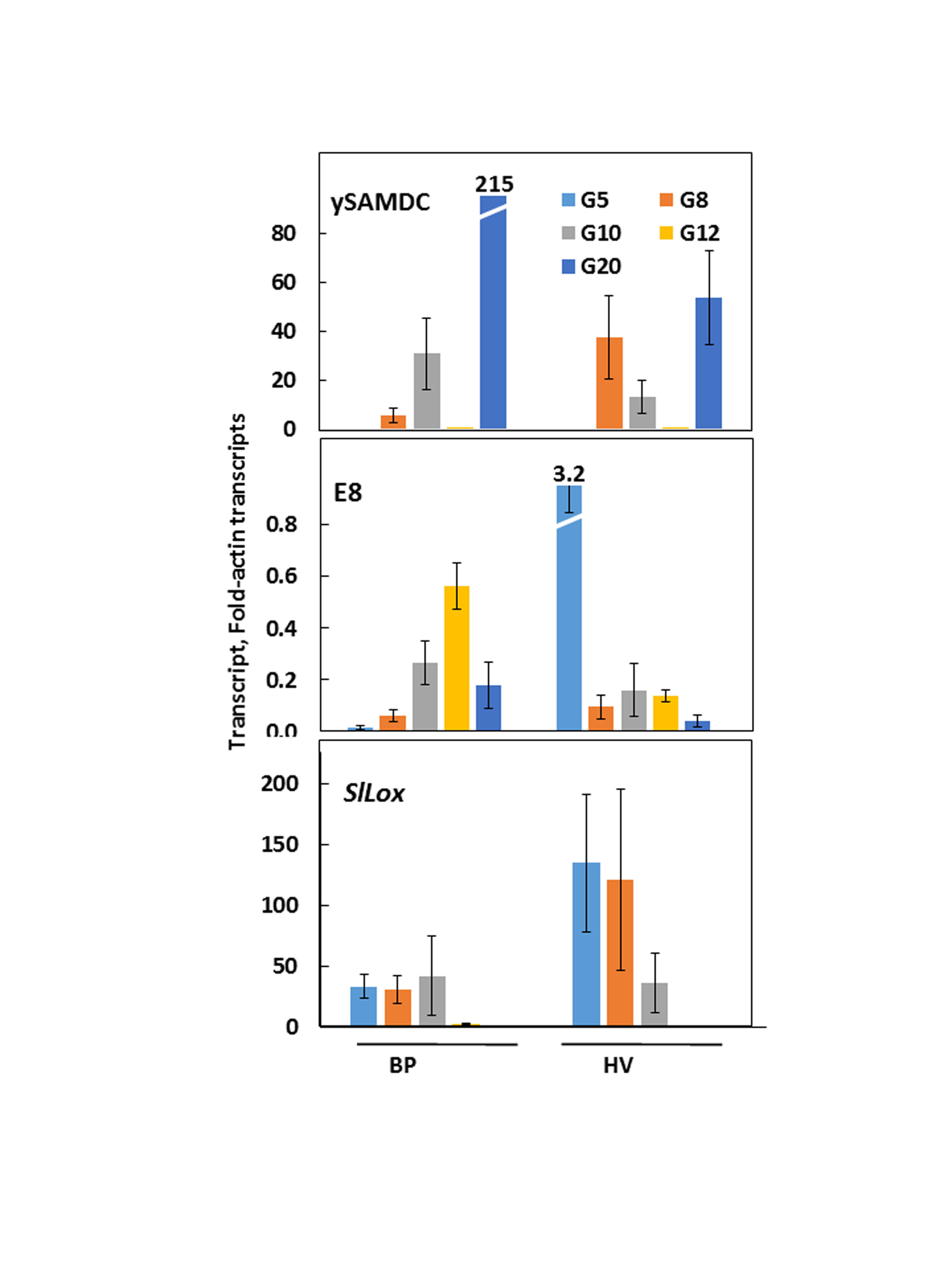


**Supplementary Fig. 1.** Relative abundance of transcript of yeast S-adenosylmethionine decarboxylase (*ySAMDC*)*,* fruit ripening gene promoter E8 (*SlE8*) and lipoxygenase (*SlLOX*) in azygous control line 5 (G5), SAMDC transgenic lines 8 and 10 (G8 and G10), lox-suppressed line 12 (G12), and line 20 (G20) - a genetic cross between line 8 and line 12. All genotypes were homozygous for the introduced transgene(s). Q-RTPCR was used to determine the transcript accumulation compared to a tomato actin gene (accession number AB199316**)** in fruit from plants grown in either black polyethylene (BP) or hairy vetch (HV) mulch. Values represent average of 4 biological replicates (combined data of pink and red stage fruit) for each data bar. Tomato genotype codes are defined in Supplementary Table 1.


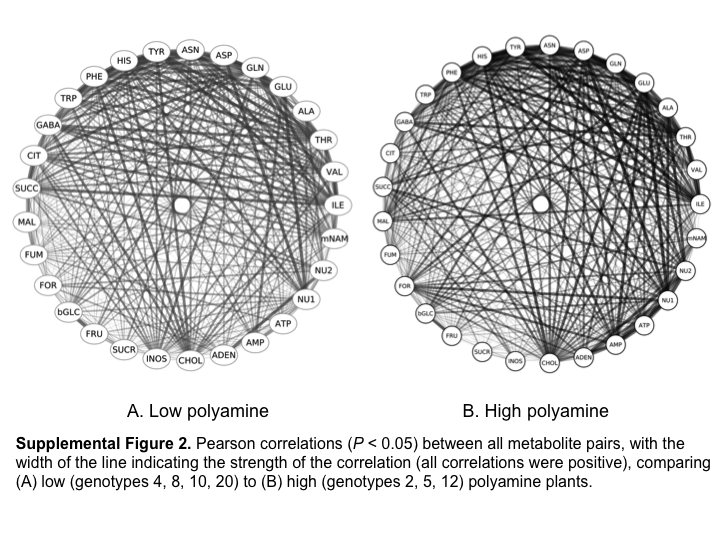


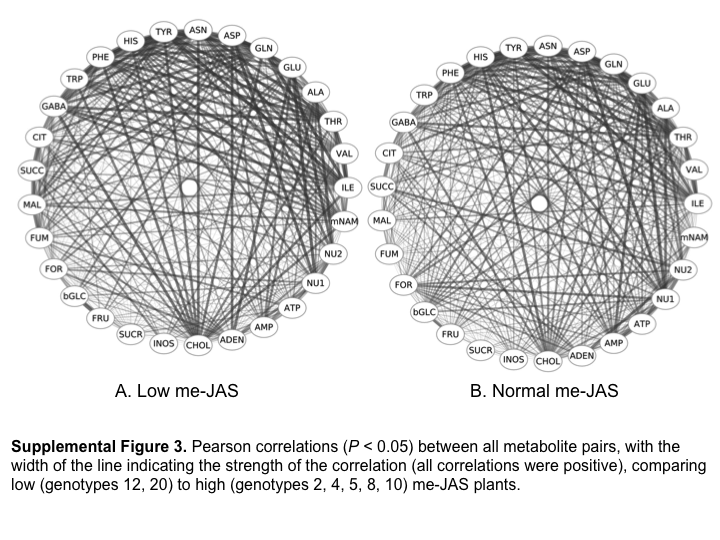


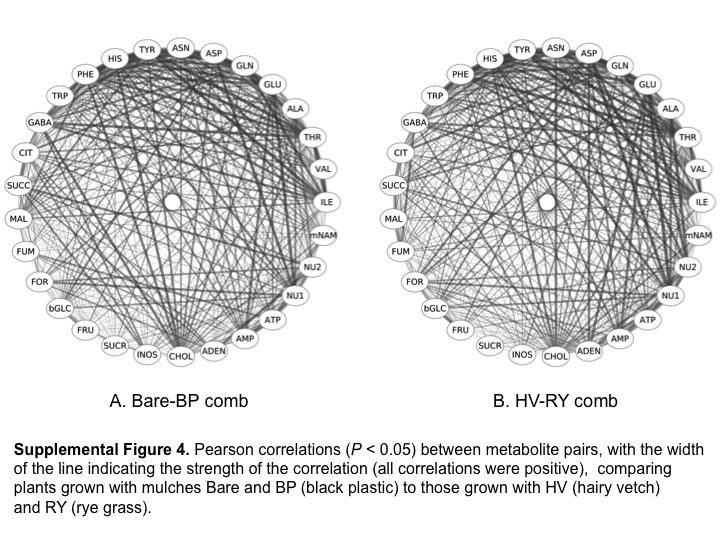

Supplement: Supplementary file 1 — Supplementary material 1 (DOC 1623 kb) [file 11306_2016_1037_MOESM1_ESM.doc]
